# A Mouse Photoreceptor Proteome Resource Identifies PALS2/MPP6 as a Novel Pan-Cone Photoreceptor Marker

**DOI:** 10.1167/iovs.66.15.44

**Published:** 2025-12-15

**Authors:** Uwe Thorsten Lux, Kerstin Reim, Johannes Leonhard Krupp, Lars Piepkorn, Elena Rudashevskaya, Olaf Jahn, Johann Helmut Brandstätter

**Affiliations:** 1 Department of Biology, Division of Animal Physiology/Neurobiology, Friedrich-Alexander-Universität Erlangen-Nürnberg, Erlangen, Germany; 2 Department of Molecular Neurobiology, Max Planck Institute for Multidisciplinary Sciences, Göttingen, Germany; 3 Neuroproteomics Group, Department of Molecular Neurobiology, Max Planck Institute for Multidisciplinary Sciences, Göttingen, Germany; 4 Department of Psychiatry and Psychotherapy, University Medical Center Göttingen, Georg-August-University, Göttingen, Germany

**Keywords:** rods, cones, mass spectrometry, retina, FACS, MPP6, PALS2, SNAP25, PSD95

## Abstract

**Purpose:**

To generate a quantitative proteome expression resource of rod and cone photoreceptors from the mouse retina that allows the exploration of cell type–specific proteomic landscapes.

**Methods:**

Fluorescence-activated cell sorting of rod and cone photoreceptors was followed by in-solution digestion with trypsin using filter-aided sample preparation. Mass spectrometry analysis was performed using data-independent acquisition (DIA).

**Results:**

Demonstrating the potential of our proteome resource, we identified PALS2 (protein associated with LIN7 2), also known as MPP6 (membrane palmitoylated protein 6), as a protein present in cone photoreceptors but absent in rod photoreceptors. We established PALS2/MPP6 as a novel pan-cone photoreceptor marker. Importantly, the high protein sequence coverage of DIA mass spectrometry allows one to gain deeper insights into photoreceptor type–specific protein isoforms. We show this with examples of PALS2/MPP6 and different synaptic proteins.

**Conclusions:**

Our quantitative proteome resource provides comprehensive details on the protein composition of mouse rod and cone photoreceptors and identifies PALS2/MPP6 as a novel pan-cone photoreceptor marker.

Photoreceptors are highly specialized light-sensing neurons in the retina that initiate visual perception by converting light into an electrical response. As ambient light levels span approximately 11 orders of magnitude, the visual system must combine high sensitivity with extreme dynamic range. For this reason, most vertebrates have a duplex retina equipped with rod photoreceptors, which respond to single photons and are optimized for scotopic and night vision, as well as cone photoreceptors, which are optimized for daylight and color vision.^1^^–^^3^ The distinct functional features of these two photoreceptor types are reflected in their different structural and molecular organization.^4^ Not only do rod and cone photoreceptors possess different visual pigments with distinct spectral properties,^5^ they are also equipped with distinct sets of proteins that play a role in various cellular processes, such as phototransduction,^6^^,^^7^ light adaptation,^8^^,^^9^ and synaptic function.^10^ Dysregulation of single proteins can lead to photoreceptor dysfunctions ranging from visual impairment to photoreceptor degeneration and blindness, as seen in various retinal diseases, including retinitis pigmentosa,^11^ retinal detachment,^12^ Leber congenital amaurosis type 12,^13^ and other retinopathies.^14^ Although they are rare, synaptopathies caused by mutated proteins at the photoreceptor ribbon synapses can also cause visual impairment and photoreceptor degeneration.^15^^–^^20^ Since rod and cone photoreceptors are often affected with varying severity, understanding the molecules and signaling pathways involved in disease development is essential.

The mouse retina is a genetically accessible model system for studying retinal function and disease. Over the past decade, single-cell transcriptomics (scRNA-seq) has revolutionized the classification of the different cell types in mouse retina.^21^^–^^24^ A recently published comprehensive single-cell atlas of the mouse retina describes 138 different cell types and presents a valuable resource for evaluating cell type–specific gene expression.^25^ However, mRNA abundance does not necessarily reflect protein abundance,^26^^,^^27^ and protein expression correlates better with disease progression and severity than gene expression.^28^ Obtaining cell type–specific quantitative proteomes from the retina is technically challenging due to the presence of many cell types. Consequently, most proteomic studies of the retina were conducted using whole retinal tissue,^28^^–^^30^ isolated photoreceptor outer segments,^31^^,^^32^ purified photoreceptor ciliary complexes,^33^ or retinal layer–specific samples.^34^^,^^35^

Here, we present the first quantitative proteome expression resource of mouse rod and cone photoreceptors. It provides comprehensive details on the protein (isoform) composition in the two types of photoreceptors. This makes it a valuable research tool for studying the biology and pathology of retinal photoreceptors.

## Materials and Methods

### Animals

Mice were group housed at the animal care facility of the Friedrich-Alexander-Universität Erlangen-Nürnberg (Biologisch-technisches Entwicklungslabor) under a 12-hour/12-hour light/dark cycle with food and water provided ad libitum. Killing of the mice to obtain retinal tissue was in accordance with the ARVO Statement for the Use of Animals in Ophthalmic and Vision Research and approved by the local authorities (Sachgebiet Tierschutzangelegenheiten der Friedrich-Alexander-Universität Erlangen-Nürnberg, AZ TS 7/2023 Tierphysiologie). Adult (aged 2–4 months) male and female C57BL/6J wild-type mice were used. For fluorescence-activated cell sorting (FACS) experiments, Tg(Rac3-EGFP)JZ58Gsat/Mmcd (Rac3-eGFP) mice, expressing enhanced green fluorescent protein (eGFP) in all cone photoreceptor cells, were used.^36^^,^^37^ These mice were obtained from the Mutant Mouse Regional Resource Center (MMRRC), a National Center for Research Resources/National Institutes of Health (NIH)–funded strain repository, and were donated to the MMRRC by the GENSAT BAC transgenic project funded by the National Institute of Neurological Disorders and Stroke.

### Fluorescence-Activated Cell Sorting of Photoreceptor Cells

**Figure 1.**
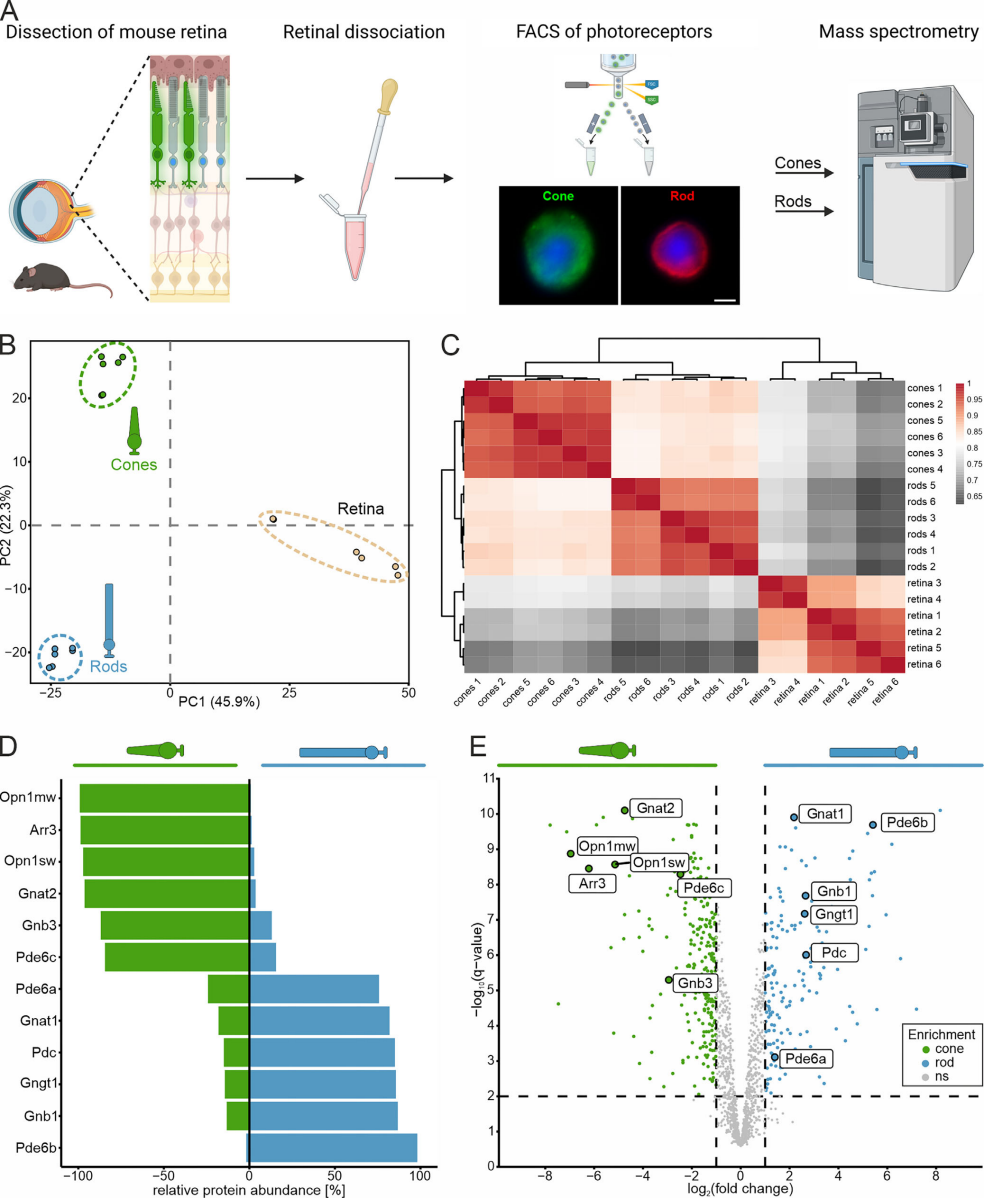
Generation and validation of a rod and cone photoreceptor proteome resource. (**A**) Schematic illustration of the FACS-based enrichment approach of rod and cone photoreceptors for mass spectrometry. Confocal micrographs show cone (anti-GFP) and rod photoreceptors (anti-Cplx4) subsequent to sorting. Cell nuclei were stained with DAPI. *Scale bar*: 2 µm. (**B**, **C**) Unbiased quality control of mass spectrometry data. Principal component analysis (**B**) and correlation heatmap (**C**) of each of the six samples for cone and rod photoreceptors and total retina homogenate. (**D**, **E**) Validation of enrichment using rod and cone photoreceptor–specific markers. Divergent bar chart (**D**) and volcano plot (**E**) of differentially enriched cone (*green*) and rod photoreceptor marker proteins (*blue*). Relative protein abundance (%) = maximum average abundance (ppm) / (average abundance cones [ppm] + average abundance rods [ppm]). Negative values represent cone enrichment; positive values represent rod enrichment. **A** created in Biorender.com. Gene names were used in **D** and **E** instead of protein names to improve readability.

Photoreceptors were sorted as described previously.^37^ Briefly, retinae of Rac3-eGFP mice were dissociated by papain digestion and subsequent trituration. Cone photoreceptors were sorted by cone photoreceptor–specific fluorescence of Rac3-eGFP mice. For the sorting of unlabeled rod photoreceptors, we used forward/sideward scatter caused by the high backscatter of heterochromatin in the core of the rod photoreceptor nucleus in an approach adapted from Feodorova et al.^38^ Rod and cone photoreceptor cells were sorted in a FACS Aria III (BD Biosciences, San Jose, CA, USA) with an 85-µm nozzle into 350 µL of predefined detergent solution (final concentration after sorting: 0.5% CHAPS and 0.1% SDS in 10 mM PBS/5 mM EDTA). After FACS sorting, the samples were allowed to reach room temperature (RT) before solid urea was added to a final concentration of 8 M. For Figure 1, images were created using FlowJo Software version 10.8.1 for Windows (Becton, Dickinson and Company, Ashland, OR, USA) and arranged with CorelDRAW 2021 (Corel Corporation, Ottawa, Canada).

### Proteome Analysis of Sorted Photoreceptors

FACS samples in lysis buffer were incubated in a cooled ultrasonic bath for 3 minutes, followed by a freeze (−20°C)/thaw cycle. This treatment was performed three times in total. Solubilized proteins were applied to centrifugal filter units (30 kDa MWCO; Merck Millipore, Darmstadt, Germany) and subjected to in-solution digestion with trypsin according to a filter-aided sample preparation (FASP) protocol as described.^39^^,^^40^ Tryptic peptides were recovered by centrifugation and extracted with 40 µL of 50 mM ammonium bicarbonate and 40 µL of 1% trifluoroacetic acid. For quantification according to the TOP3 approach,^41^ combined flow-throughs were spiked with 10 fmol/µL Hi3 *Escherichia coli* standard (Waters Corporation, Eschborn, Germany; contains a set of quantified synthetic peptides derived from *E. coli* chaperone protein ClpB) and directly subjected to liquid chromatography–mass spectrometry (LC-MS) analysis. Nanoscale reversed-phase ultra-performance liquid chromatography (UPLC) separation of tryptic peptides was performed over 150 minutes at a flow rate of 300 nL/min in an increasing gradient of mobile phase B comprising two linear steps: 3% to 35% for 135 minutes, followed by 35% to 60% for 15 minutes. Gradient was formed by mixing mobile phase A (0.1% formic acid in water) and mobile phase B (0.1% formic acid in acetonitrile). MS analysis on a quadrupole time-of-flight mass spectrometer (Synapt G2-S/-Si; Waters Corporation) was performed in the ion mobility-enhanced data-independent acquisition (DIA) mode with drift time–specific collision energies, referred to as UDMS^E^.^42^

### Analysis of Proteome Data

The continuum LC-MS data generated as described above were processed using the Waters ProteinLynx Global Server and searched against a custom database compiled by adding the sequences of *E. coli* chaperone protein ClpB (UniProtKB/Swiss-Prot accession number P00924) and porcine trypsin (P00761) to the UniProtKB/Swiss-Prot mouse proteome (release 2024-03, 17,217 entries). As additional retina-specific adaptations of the database, the sequences of the established retina protein isoforms, isoform 3B of syntaxin 3 (Q64704-2), isoform 2 of C-terminal binding protein 2 (RIBEYE, P56546-2), and isoform 2 of disks large homolog 4 (PSD95β, Q62108-2), were used to replace the respective canonical isoform. Furthermore, the sequences of isoform alpha of protein PALS2 (Q9JLB0-2) and isoform 2 of synaptosomal-associated protein 25 (P60879-2) were added. The reversed sequence of each entry was appended to the sequence file to enable the determination of the false discovery rate (FDR) set to 1% threshold.

For postidentification analysis, including TOP3 protein quantification, the freely available software ISOQuant^42^ was used as described.^39^^,^^43^ Only proteins represented by at least two peptides (minimum length six amino acids, score ≥5.5, identified in at least two runs) were quantified as parts per million (ppm) (i.e., the relative amount (w/w) of each protein with respect to the sum over all detected proteins). FDR for both peptides and proteins was set to 1% threshold, and at least one unique peptide was required. Proteins identified as contaminants from blood (albumin, hemoglobin) or skin/hair cells (keratins) were removed, and potential outlier proteins were revised by inspecting the quality of peptide identification, as well as the quantification and distribution between protein isoforms. Filtered protein lists were log_2_-transformed, and groupwide missing values (e.g., when a protein has no data in one of the groups) were replaced by low, random values based on the statistical distribution of each individual sample. For this purpose, the mean and standard deviation of the observed intensity values were calculated for each sample to obtain the center and width of its distribution (A), denoted as µ_A_ and σ_A_. Random values were then drawn from a normal distribution (B) with a down-shifted center at µ_B_ = µ_A_ − 3.0 × σ_A_ and a reduced width of σ_B_ = 0.3 × σ_A_. Imputed data were subjected to statistical analysis with the Bioconductor R packages “limma” and “q-value” (Supplementary Table S7) to calculate the log_2_ fold-change (FC) and the significance of differences for protein abundance alterations by moderated *t*-statistics as described.^39^^,^^43^ The mass spectrometry proteomics data have been deposited to the ProteomeXchange Consortium via the PRIDE^44^ partner repository with the data set identifier PXD066755.

For reanalysis of PRIDE/ProteomeXchange data set PXD034057,^35^ Proteome Discoverer 3.1 (version 3.1.0.638; Thermo Fisher Scientific, Waltham, MA, USA) was used to search the downloaded raw data against the customized protein sequence database described above. Sequest HT search engine settings were as in Todorova et al.^35^: full trypsin (with no cleavage at Pro), two missed cleavages allowed, precursor mass tolerances set to 10 ppm, and fragment mass tolerance of 0.02 Da. Static modifications included carbamidomethylation of Cys; dynamic modifications included oxidation of Met and deamidation of Asn and Gln. Also, loss of Met and acetylation at the protein N-terminus were allowed. Validation was made with Percolator, the posterior error probability (PEP) value was set as a base for validation, and only high confidence identifications with FDR <1% were accepted. A strict parsimony principle was used for protein grouping to create the list of identified proteins.

### Bioinformatic Analyses

Proteome data were analyzed and visualized in an R statistical environment^45^ using the packages summarized in Supplementary Table S7. All scripts used for visualization were generated in RStudio (Posit PBC, Boston, MA, USA) and are available as annotated R Notebooks in the supplementary information. The Mouse Retina Cell Atlas (MRCA) scRNA-seq data set^25^ was downloaded as an AnnData object from the CELLxGENE data collection at https://cellxgene.cziscience.com/collections/a0c84e3f-a5ca-4481-b3a5-ccfda0a81ecc (May 24, 2024), processed in a Python environment using scanpy^46^ and analyzed using dedicated Python and R packages (Supplementary Table S7). Expression levels of selected genes were grouped by major cell classes, which were clustered according to the authors. For pseudobulk analysis, the MRCA data set was reduced to consist only of photoreceptors, and data sets with a low photoreceptor count and quality were excluded. Photoreceptor pseudobulks were aggregated by summation of raw counts within groups defined by sample and cell type, resulting in photoreceptor type–specific biological replicates. The pseudobulk count matrix and metadata were imported into R for differential gene expression analyses with DESeq2.^47^ Generated graphs were arranged using CorelDRAW 2022 (Corel Corporation).

### Immunocytochemistry

Preparation of mouse retinae for cryostat sections and antibody incubation for light microscopic immunocytochemistry were done as described previously with minor modifications.^37^ Briefly, mice were deeply anesthetized by inhalation of isoflurane (Abbott Laboratories, Chicago, IL, USA) and killed by cervical dislocation. The eyes were opened and fixed in the eyecup in 4% paraformaldehyde in PBS (10 mM, pH 7.4) for 30 minutes at RT. After washing, the eyes were cryoprotected in 10%, 20% and 30% (w/v) sucrose in 10 mM PBS and mounted in Tissue-Tek O.C.T. freezing medium (Sakura Finetek Germany, Staufen, Germany). Vertical cryostat sections (14 µm thick) were cut with a cryostat (CM3050 S; Leica Microsystems, Wetzlar, Germany) and collected on glass slides. For the immunocytochemical experiments with sorted photoreceptors, the cells were sorted into 350 µL 10 mM PBS and immediately transferred to poly-D-lysine–coated glass slides. The glass slides containing photoreceptors or retinal sections were then washed in 10 mM PBS and blocked in blocking solution (10% normal goat or donkey serum [NGS/NDS], 1% bovine serum albumin [BSA], 0.5% Triton X-100 in 10 mM PBS) for 1 hour at RT. Primary antibodies were diluted in antibody solution (3% NGS/NDS, 1% BSA, 0.5% Triton X-100 in 10 mM PBS), and incubation was performed overnight at 4°C. Zenon labeling technology (Thermo Fisher Scientific) was used for generating A488 (cat. #Z25302) and A647 (cat. #Z25308) primary antibody conjugates. The next day, the samples were washed three times with 10 mM PBS and incubated with secondary antibodies and DAPI (0.1 µg/mL) diluted in antibody solution for 2 hours at RT. After final washing steps with 10 mM PBS, the samples were mounted using Aqua Polymount (Polysciences, Warrington, PA, USA).

### Antibodies

The following primary antibodies were used for immunocytochemistry: guinea pig anti–calbindin D28k (1:2000; cat. #214 005; Synaptic Systems GmbH, Göttingen, Germany), mouse anticalretinin (1:2000, cat. #MAB1568; Chemicon, Temecula, CA, USA), mouse anti–complexin 3 (1:500, cat. #122 311; Synaptic Systems GmbH), rabbit anti–complexin 4 (1:40,000; cat. #122 402; Synaptic Systems GmbH), mouse anti-GFP (1:1000, cat. #A-11122; Invitrogen/Thermo Fisher Scientific), rabbit anti–cone arrestin (1:750 Zenon-labeled; Chemicon), rabbit anti–M-opsin (1:300 Zenon-labeled, cat. #AB5407; Chemicon), rabbit anti–membrane palmitoylated protein 4 (1:5000, cat. #220 103; Synaptic Systems GmbH), rabbit anti–membrane palmitoylated protein 6 (1:5000, 1:1000 Zenon-labeled, cat. #USC-PAA700HU01; Cloud-Clone, Houston, TX, USA), mouse anti–protein kinase C alpha (1:20,000; cat. #610107; BD Transduction Laboratories, Franklin Lakes, NJ, USA), rabbit antisecretagogin (1:2000 Zenon-labeled, cat. #BVD-RD181120100; BioVendor, Brno, Czech Republic), goat anti–S-opsin (1:1000, cat. #ab235274; Abcam, Cambridge, UK), and guinea pig anti–vesicular glutamate transporter 1 (1:50,000; cat. #AB5905; Chemicon).

Fluorophore-coupled secondary antibodies were used for visualization of primary antibodies: Alexa Fluor Plus 488/555/647–conjugated goat antimouse, antirabbit, and anti–guinea pig IgG (1:200–1:500; cat. #A21422, A21435, A32728, A32731, A32732, A32733; Thermo Fisher Scientific) and Alexa Fluor Plus 405/568–conjugated donkey antirabbit and donkey antigoat IgG, respectively (1:500; cat. #A48258, A11057; Thermo Fisher Scientific). Cell nuclei were labeled with DAPI (0.1 µg/mL).

### Light Microscopy and Analysis of Immunofluorescence Data

For light microscopical analysis, labeled sections were examined with an Axio Imager.M2 equipped with an ApoTome.2 module or a Laser Scanning Microscope 710 with corresponding imaging modules (Carl Zeiss AG, Oberkochen, Germany). Images were acquired using a 20× (0.8 NA, Apochromat) or a 63× (1.4 NA oil immersion, Plan Apochromat) objective (both Carl Zeiss AG) as stacks of multiple optical sections and projections were calculated with ZEN blue or ZEN black software (Carl Zeiss AG). Images were adjusted for contrast and brightness using ZEN blue (Carl Zeiss AG) and arranged using CorelDRAW 2022 (Corel Corporation). Fluorescence intensities of line profiles were measured using the ImageJ distribution Fiji.^48^ The obtained information was summarized in Excel (Microsoft, Redmond, WA, USA), and fluorescence intensities were normalized to maximum values. Graphs were created using GraphPad Prism 10.4.2 (GraphPad Software, San Diego, CA, USA) and arranged using CorelDRAW 2022 (Corel Corporation).

## Results

### Quantitative Mass Spectrometry Analyses of Sorted Rod and Cone Photoreceptors Demonstrate Distinct Proteome Profiles

Our goal was to create a resource of the proteome of rod and cone photoreceptors in the mouse retina that can serve as a tool for photoreceptor type–specific identification and assignment of proteins. To this end, we optimized our previously established trituration-based FACS strategy^37^ to enrich mouse rod and cone photoreceptors for mass spectrometry analysis (Fig. 1A). Due to the cell isolation and sorting process, the photoreceptors do not retain their native morphology, resulting in an enrichment of mainly perikarya (Fig. 1A). For seamless interfacing of FACS with LC-MS, photoreceptors were directly sorted into a predefined detergent-containing buffer, to which solid urea was added after completion of sorting. The resulting protein samples in 0.5% CHAPS/0.1% SDS/8 M urea (final concentrations) could be readily subjected to tryptic in-solution digestion according to the FASP protocol.^40^^,^^49^ We obtained rod and cone photoreceptor samples from six male mice independently (Supplementary Table S1), processed them as pools of two for FASP, and performed LC-MS analyses of the respective three biological replicates with replicate injections, allowing us to perform statistical analyses of the resulting six data sets per condition. Dissociated whole retina (i.e., the input for the FACS procedure) was processed in parallel to be able to assess the enrichment of cone and rod photoreceptor–associated proteins in the respective fractions, rather than to provide a deep retina proteome (i.e., we refrained from using prefractionation and longer separation gradients).

By ion mobility-enhanced DIA MS, we identified 1556 proteins with an average sequence coverage of 32.8% in our retina lysate and rod and cone photoreceptor samples (Supplementary Data S1) and analyzed their expression profiles using bioinformatic tools. Figure 1B shows the clustering of total retina and the rod and cone photoreceptor samples in different regions of the principal component analysis, which indicates a similar protein enrichment in all three biological replicates of the same sample type and in general a close clustering of the technical replicates. The correlation heatmap (Fig. 1C) agrees well with these findings and illustrates a high correlation between samples of the same type (i.e., rod photoreceptors, cone photoreceptors, and retina). Furthermore, the heatmap shows that the proteomic profiles of rod photoreceptors are more similar to cone photoreceptor proteomic profiles than to total retina profiles, indicating an enrichment of photoreceptor-specific proteins.

To validate the specificity of our mouse photoreceptor proteome resource, we checked for potential cross-contamination with other retinal cell types using well-established cell type–specific markers: calbindin (CALB1) for horizontal cells, calcium binding protein 5 (CABP5) for rod and cone bipolar cells, paired box 6 (PAX6) for amacrine cells, RNA binding protein with multiple splicing (RBPMS) for ganglion cells, and glial fibrillary acidic protein (GFAP) for glial cells (for additional markers, see Supplementary Table S2). We did not find these neuronal cell-type markers in our proteome data, which we interpreted as an indicator of efficient photoreceptor enrichment. However, we cannot fully exclude the possibility that apparent sensitivity limitations may have prevented the mass spectrometric identification of these presumably low-abundant proteins, given that their detectability has recently been shown in an ultra-deep proteome data set from bulk retina.^30^ In contrast, GFAP was clearly identified in all LC-MS runs of all three fractions: whole retina and rod and cone photoreceptor samples (Supplementary Data S1; Supplementary Table S2). Müller glia cells are essential for the function and viability of photoreceptors and other retinal neurons, which they tightly ensheath.^50^^,^^51^ This is likely the reason why glial cell fragments were present in our sorted photoreceptor samples. Notably, the retina is a complex and tightly associated neuronal tissue, and absolute purity of any photoreceptor type through cell sorting is not possible. Nevertheless, our assessment of cross-contamination indicates that most of the identified proteins originate from photoreceptors and Müller glia cells.

Next, we examined the presence of well-characterized cone and rod photoreceptor markers in our proteome data to verify their quality further (Figs. 1D, 1E). The divergent bar chart in Figure 1D illustrates the relative protein abundances and indicates a strong enrichment in the respective samples. The volcano plot in Figure 1E shows that cone and rod photoreceptor markers are well separated and restricted to the upper left and right corners, respectively. In summary, all photoreceptor markers were significantly enriched in the respective photoreceptor samples, with a −log_10_[*q*-value] > 2 (corresponding to *q* < 0.01) and a log_2_FC > |1| (corresponding to twofold enrichment), which confirms the quality and specificity of our proteome resource.

### Overrepresentation Analyses Highlight Energy Demand of Cone Photoreceptors

**Figure 2.**
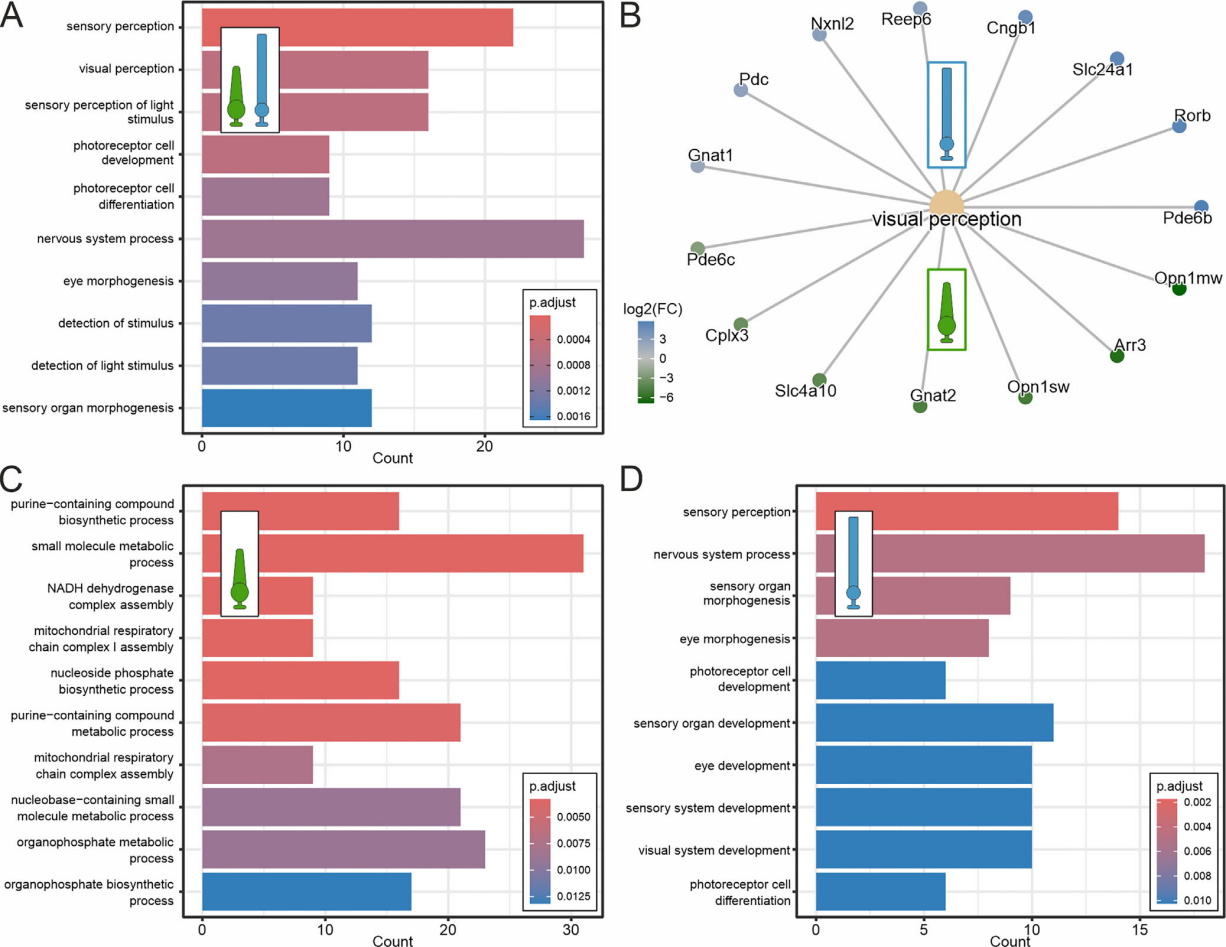
ORAs of rod and cone photoreceptor–enriched proteins highlight energy demand of cones. (**A**) Bar plot of the top 10 enriched biological pathways in both photoreceptor types using ORA. (**B**) Circular network plot of significantly enriched rod and cone photoreceptor proteins of the visual perception pathway. Gene names were used instead of protein names to improve readability. (**C**, **D**) Bar plots of the top 10 enriched biological pathways in cone (**C**) and rod photoreceptors (**D**). Proteins were considered significantly enriched for ORA if −log_10_[*q*-value] > 2 and log_2_FC < −2 (cones) and/or log_2_FC > 2 (rods).

To gain insights into the most relevant photoreceptor proteins and pathways, we conducted overrepresentation analyses (ORAs). First, we analyzed the proteins showing significant (−log_10_[*q*-value] > 2) and strong (log_2_FC > |2|) enrichment in both photoreceptor types (Figs. 2A, 2B; Supplementary Data S2). As expected, the most represented biological pathways are related to visual perception (Fig. 2A), and many of the enriched proteins are key components of the phototransduction cascade and exhibit photoreceptor-specific distribution (Fig. 2B; Supplementary Table S3). Second, we investigated the differences in overall biological pathway representation between rod and cone photoreceptors. To do so, we performed independent cone photoreceptor–specific (log_2_FC < −2) and rod photoreceptor–specific (log_2_FC > 2) ORAs (Figs. 2C, 2D; Supplementary Data S2). While the most significant biological pathways in rod photoreceptors were also related to sensory perception (Fig. 2D), we identified numerous pathways in cone photoreceptors associated with biosynthesis, energy metabolism, and mitochondrial processes (Fig. 2C). These findings agree well with published data, showing that a single cone photoreceptor requires approximately twice the energy of a single rod photoreceptor.^52^ Thus, the results corroborate our proteome, demonstrating its versatility in characterizing photoreceptor-specific molecular networks at the protein level.

### Comparative Mouse Photoreceptor Proteome: Insights Into the Synaptic Vesicle Release Machinery of Rod and Cone Photoreceptors

Next, we verified the validity of our proteome resource for analyzing proteins of interest in the two types of photoreceptors. We focused on the regulation of synaptic vesicle exocytosis by the SNARE complex at the photoreceptor ribbon synapse. This is a unique type of chemical synapse present in both rod and cone photoreceptors. Ribbon synapses enable the continuous release of the neurotransmitter glutamate in the dark and the gradual change in release rate with changing light intensities.^53^^,^^54^

**Figure 3.**
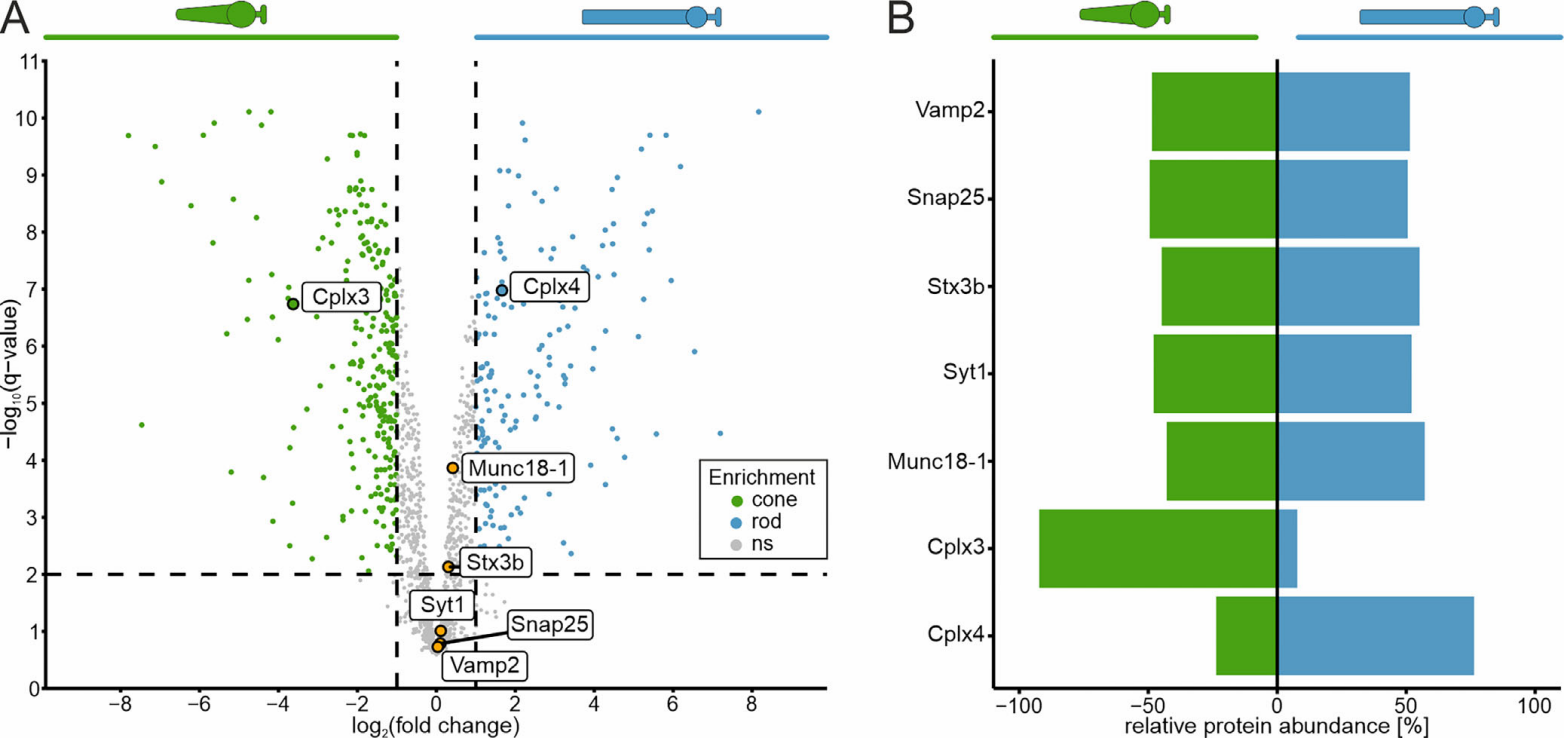
Distribution of SNARE complex components and SNARE-regulating complexins (Cplx) in photoreceptors of the mouse retina. (**A**) Volcano plot of differentially enriched proteins in cone (*green*) and rod photoreceptors (*blue*). SNARE complex components (Vamp2, Snap25, Stx3), the essential modulator of the synaptic vesicle fusion protein complex Munc18-1, and the calcium sensor Syt1 are highlighted in *orange*. Cplx3 and Cplx4 are highlighted in *green* (cone enriched) and *blue* (rod enriched), respectively. (**B**) Divergent bar chart. Relative protein abundance (%) = maximum average abundance (ppm) / (average abundance cones [ppm] + average abundance rods [ppm]). Negative values represent cone enrichment; positive values represent rod enrichment. Gene names were used instead of protein names to improve readability.

The volcano plot shows that the proteins of the core complex of SNARE-dependent synaptic vesicle fusion were not differentially enriched in the two photoreceptor types (Fig. 3). These include VAMP2 (synaptobrevin 2), SNAP25, and syntaxin 3B (STX3B),^55^^–^^58^ as well as the essential component of the synaptic vesicle fusion protein complex MUNC18-1 (mammalian uncoordinated-18)^59^ and the calcium sensor synaptotagmin 1 (SYT1).^60^^,^^61^ Given the relatively high protein abundance levels (Supplementary Data S1), our data confirm previous findings, reporting that the ribbon synapses of cone and rod photoreceptors are equipped with MUNC18-1^62^ and SYT1^63^ and comprise the same core SNARE complex consisting of VAMP2, SNAP25, and STX3B instead of STX1.^57^^,^^58^

Regulators of SNARE-dependent synaptic vesicle fusion are the complexins (CPLXs), small proteins that bind to the assembled SNARE complex, thereby regulating exocytosis.^64^^,^^65^ All four known CPLX isoforms are expressed in the mouse retina, but only CPLX3 and CPLX4 are found at the ribbon synapses.^10^ In a previous study, we demonstrated at both the transcript and protein level that cone photoreceptors express CPLX3 and CPLX4, whereas CPLX4 is the predominant CPLX isoform in rod photoreceptors.^37^ Figure 3A shows a significant enrichment of CPLX3 in cone and CPLX4 in rod photoreceptors. An assessment of the protein abundance further clarifies that CPLX3 was barely detected in rod photoreceptors. In contrast, CPLX4 exhibited a high abundance in both types of photoreceptors, though it was approximately three times more abundant in rod photoreceptors (Fig. 3B; Supplementary Data S1).

Taken together, these results demonstrate that our mouse photoreceptor proteome resource can be used to characterize protein expression in cone and rod photoreceptors.

### Identification of Protein Associated With LIN7 2 (PALS2) as a Novel Pan-Cone Photoreceptor Protein

**Figure 4.**
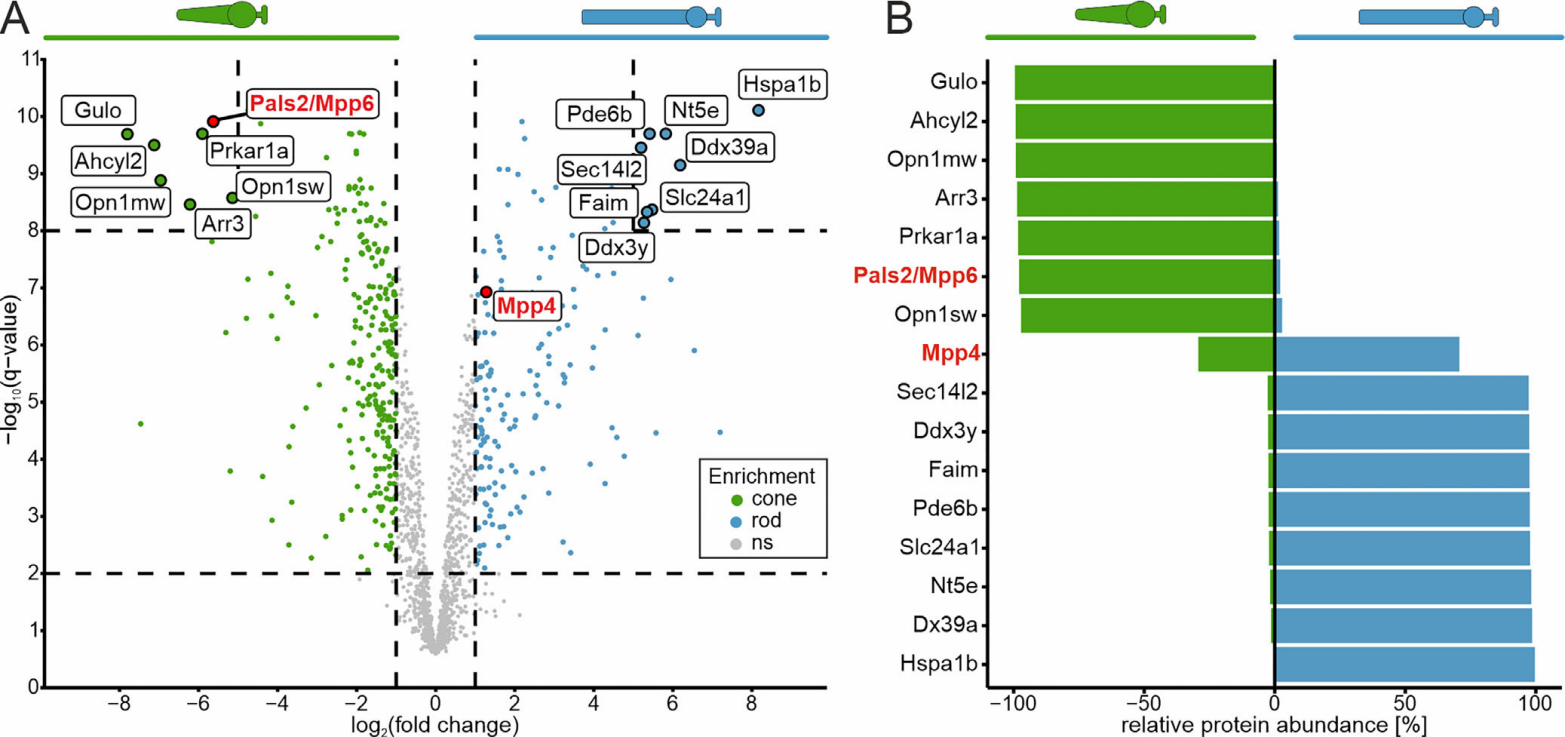
Pals2/Mpp6 is highly enriched in cone photoreceptors. (**A**) Volcano plot of differentially enriched proteins in cone (*green*) and rod photoreceptors (*blue*). Highlighted are highly enriched proteins with −log_10_[*q*-value] > 8 and log_2_FC < −5 (cones, *green*) and log_2_FC > 5 (rods, *blue*), as well as Pals2/Mpp6 and Mpp4 (*red*). (**B**) Divergent bar chart. Relative protein abundance (%) = maximum average abundance (ppm) / (average abundance cones [ppm] + average abundance rods [ppm]). Negative values represent cone enrichment; positive values represent rod enrichment. Gene names were used instead of protein names to improve readability.

Intrigued by the opportunity to use our mouse photoreceptor proteome resource to identify novel cone or rod photoreceptor–specific proteins, we determined the top candidates enriched in either cone or rod photoreceptors. By setting stringent thresholds of −log_10_[*q*-value] > 8 and log_2_FC > |5|, we selected seven and eight highly enriched proteins in cone and rod photoreceptors, respectively (Fig. 4; Supplementary Table S4). As expected, the list includes cone and rod photoreceptor–specific proteins that were already established (Figs. 1D, 1E, 4). More importantly, with PALS2 (protein associated with LIN7 2), also known as MPP6 (membrane palmitoylated protein 6),^66^ it also includes a protein that, to our knowledge, has not previously been characterized in photoreceptors (Fig. 4). Mpp proteins (MPP1–7) are a subfamily of membrane-associated guanylate kinases (MAGUKs).^66^ MAGUKs are multidomain scaffolding proteins that serve as a platform for various cellular processes, including cell polarity, adhesion, plasticity, and synapse development and signaling.^67^^–^^69^

A known MPP in the photoreceptors of the mouse retina is MPP4.^70^ Therefore, we compared the expression of MPP4 and PALS2/MPP6 in cone and rod photoreceptors. We found that MPP4 (identified with a sequence coverage of 55.0%) was slightly enriched in rod photoreceptors, whereas PALS2/MPP6 (identified with a sequence coverage of 74.1%) was highly enriched in cone photoreceptors (Fig. 4A). Closer examination of the relative protein abundance (Fig. 4B; Supplementary Data S1) revealed that MPP4 is present in both photoreceptor types (mean relative abundance of 250 ppm in cone photoreceptors vs. 603 ppm in rod photoreceptors), while PALS2/MPP6 abundance was high in cone (2416 ppm) and negligible in rod photoreceptors (52 ppm). To confirm our proteome findings at the transcript level, we performed photoreceptor-specific pseudobulk and differential expression analyses using recently published scRNA-seq data.^25^ Our results show that the Mpp4 transcript was present in both photoreceptor types, whereas the Pals2/Mpp6 transcript was restricted to cone photoreceptors (Supplementary Figs. S1, S2).

**Figure 5.**
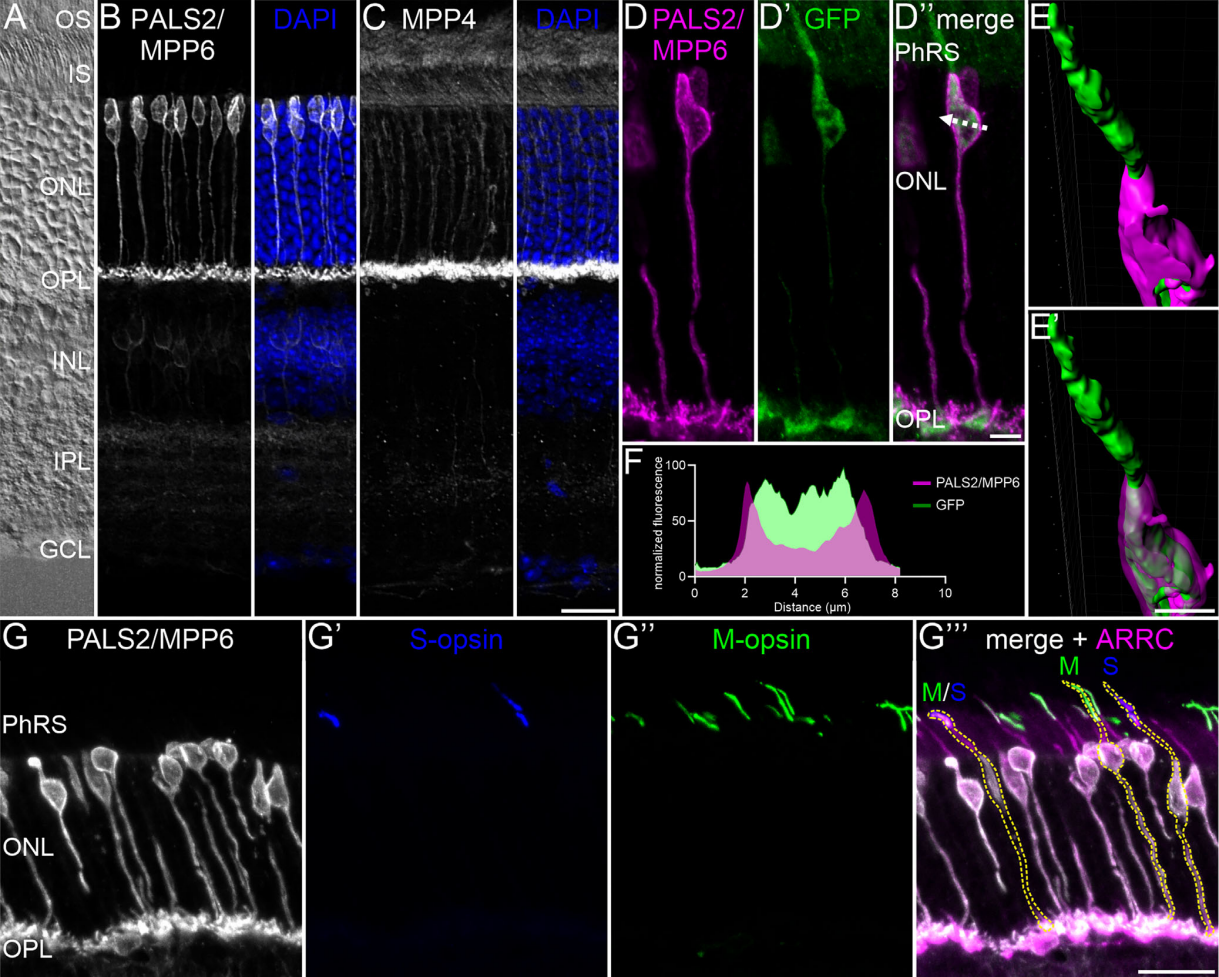
PALS2/MPP6 is a novel pan-cone photoreceptor marker. (**A**) Nomarski micrograph of a vertical cryostat section through mouse retinae showing the different retinal layers. (**B**, **C**) Fluorescence micrographs of vertical cryostat sections through mouse retinae stained with anti-PALS2/MPP6 (**B**) and anti-Mpp4 (**C**). Cell nuclei were stained with DAPI. (**D–E′**) Confocal micrograph of a vertical cryostat section through Rac3-eGFP mouse retinae, expressing GFP in cone photoreceptors, stained with anti-PALS2/MPP6 (**D**) and anti-GFP (**D′**). Both channels are displayed in the merged image (**D′′**) and in three-dimensional reconstruction (**E–E′**). Line profile through a single cone photoreceptor (**F**) as indicated by the *white dotted line* in **D′′**. (**G–G′′′**) Confocal micrographs of vertical cryostat sections through mouse retinae stained with anti-PALS2/MPP6 (**G**), anti–S-opsin (**G**′), anti–M-opsin (**G**′′), and anti–cone arrestin (ARRC). All four channels are displayed in the merged image (**G′′′**) and M-, S-, and M/S-cones are highlighted with *yellow dotted lines*. GCL, ganglion cell layer; INL, inner nuclear layer; PhRS, photoreceptor inner and outer segments. *Scale bars*: 20 µm in **C** for **A–C** and **G′′′** for **G–G′′′**; 5 µm in **D′′** for **D–D′′** and **E′** for **E–E**′.

Having identified the presence of PALS2/MPP6 in cone photoreceptors using our proteome resource, we next verified the photoreceptor type–specific expression and subcellular localization of PALS2/MPP6 with immunocytochemistry (Fig. 5). Immunocytochemical staining of vertical retinal cryostat sections using anti-PALS2/MPP6 antibodies showed strong PALS2/MPP6 labeling in the somata and axons of cone photoreceptors in the outer nuclear layer (ONL), as well as in their synaptic terminals in the outer plexiform layer (OPL) (Figs. 5A, 5B; Supplementary Fig. S3). Additionally, we observed weak PALS2/MPP6 staining in the inner nuclear layer and the inner plexiform layer (Figs. 5A, 5B), originating from horizontal cells and cone bipolar cells, respectively (Supplementary Figs. S4, S5). The gold standard for verifying the specificity of the PALS2/MPP6 antibodies would be a knockout control, but this is not available to us. Nevertheless, the immunocytochemical findings agree well with our expression analysis of previously published scRNA-seq data^25^ (Supplementary Fig. S2).

Unlike PALS2/MPP6, MPP4 staining was present only in the outer retina. Weak MPP4 staining was found in the photoreceptor outer segments (OSs) and inner segments (ISs), as well as in their somata and axons in the ONL. Strong MPP4 staining was present in the photoreceptor synaptic terminals in the OPL (Figs. 5A, 5C).

To examine the cellular localization of PALS2/MPP6 in cone photoreceptors in more detail, we double-labeled vertical retinal cryostat sections from Rac3-eGFP mice, which express eGFP in cone photoreceptors,^36^^,^^37^ using anti-GFP and anti-PALS2/MPP6 antibodies. PALS2/MPP6 staining was prominent in the GFP-positive cone photoreceptor somata and synaptic terminals but absent from cone photoreceptor outer and inner segments (Figs. 5D–E′). Fluorescence intensity measurements revealed that PALS2/MPP6 is predominantly located at the plasma membrane of cone photoreceptors (Fig. 5F), which agrees well with the described membrane targeting properties of PALS2/MPP6.^66^

Finally, labeling of vertical retinal cryostat sections with anti-PALS2/MPP6 antibodies and markers for the different types of cone photoreceptors in mouse retina revealed the presence of PALS2/MPP6 in all cone photoreceptors (Figs. 5G–G′′′).

### The Mouse Photoreceptor Proteome Resource: Validation and Exploration of Photoreceptor Type–Specific Protein Isoforms

Photoreceptor ribbon synapses comprise unique sets of proteins that differ from conventional chemical synapses.^54^ Here, we asked whether rod and cone photoreceptor ribbon synapses differ in their expression of protein isoforms and whether our photoreceptor proteome resource could distinguish between them. As proof of concept, we selected RIBEYE^71^ and STX3B^58^ as protein candidates previously described in both photoreceptor types and thoroughly analyzed the respective tryptic peptides identified in our proteome data (Supplementary Figs. S6A, S6B; Supplementary Data S3). The detection of unique tryptic peptides for the N-terminal part of RIBEYE (Supplementary Fig. S6A), as well as for the C-terminal part of STX3B (Supplementary Fig. S6B), confirms the expression of both proteins in the two photoreceptor types and validates our analysis strategy.

Next, we compared the photoreceptor type–specific abundance of isoforms of the newly identified cone photoreceptor protein PALS2/MPP6 and the synaptic proteins PSD95 and SNAP25.

**Figure 6.**
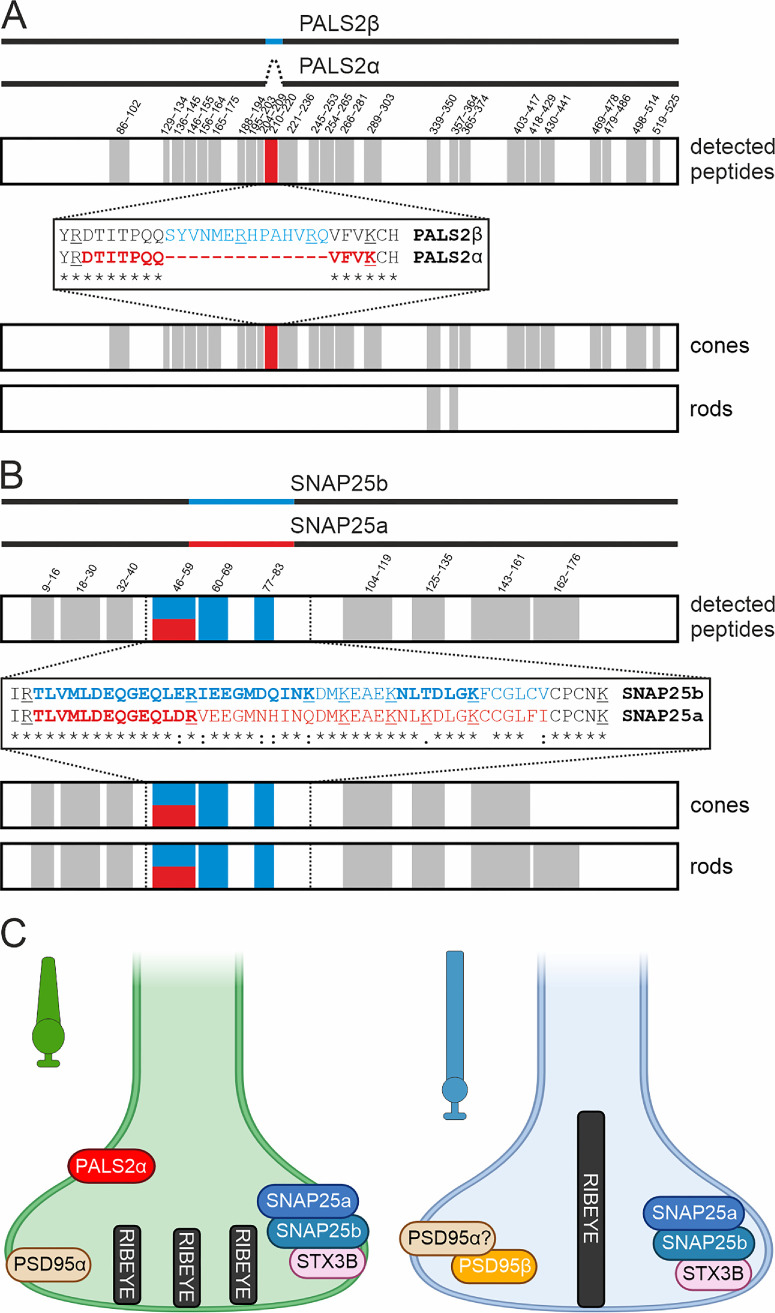
Identification of photoreceptor type–specific protein isoforms. (**A**, **B**) Identified tryptic peptides (*n* ≥ 3 per group) for PALS2α/PALS2β (**A**) and SNAP25a/SNAP25b (**B**). Peptides assigned to regions with identical amino acid sequences between the two compared isoforms are shown in *gray*; isoform-specific peptides are highlighted in *red* and *blue*. Sequence alignments display detected peptides in *bold*; tryptic cleavage sites are *underlined*. (**C**) Schematic representation of identified protein isoforms in rod and cone photoreceptors. For the corresponding peptide displays of RIBEYE, syntaxin 3b, and PSD95, see Supplementary Figure S6. **C** created in Biorender.com.

Representing a novel pan-cone photoreceptor protein, we assessed the expression of the two known isoforms of PALS2/MPP6, PALS2α, and/or PALS2β (Fig. 6A).^66^ The shorter PALS2α (539 amino acids [aa]) lacks 14 aa of the longer canonical PALS2β sequence (553 aa). While we detected several shared tryptic peptides framing the splice region, indicating that this part of the protein is well accessible for trypsin, we did not identify any tryptic peptides from the sequence stretch unique for the canonical isoform PALS2β. Instead, we confidently identified the tryptic peptide 210-DTITPQQVFVK-220 (Supplementary Table S5), which only occurs upon splicing and thus demonstrates the expression of the noncanonical PALS2α in cone photoreceptors. However, on the basis of our mass spectrometric data, we cannot fully exclude the concomitant presence of PALS2β in the (unlikely) case that corresponding isoform-specific tryptic peptides may have escaped detection.

PSD95 is presynaptically present in both photoreceptor types.^72^ N-terminal alternative splicing has been shown to generate the two isoforms PSD95α and PSD95β,^73^ both expressed at the transcript level in the retina.^74^^,^^75^ Our data confirm that PSD95 is expressed in both photoreceptors. However, we detected a unique tryptic peptide for the noncanonical isoform PSD95β only in rod (6/6 samples) but not in cone photoreceptors (0/6 samples) (Supplementary Fig. S6C; Supplementary Data S3). The identified isoform-specific tryptic peptide (10-SALWLLAPPLLR-21) matches the N-terminal L27 domain of PSD95β, via which it has previously been shown to copurify with the photoreceptor protein MPP4.^75^

In mouse brain, Snap25a is the dominant transcript during embryonic and early postnatal development, and Snap25b becomes the predominant transcript (>95%) in adulthood.^76^ Interestingly, our proteome resource revealed that both splice variants are coexpressed in rod and cone photoreceptors (Fig. 6B; Supplementary Table S6). This agrees well with the presence of both Snap25a and Snap25b at the transcript level in the adult mouse retina.^77^

In summary, we have increased our understanding of the presence of specific protein isoforms in rod and cone photoreceptors (Fig. 6C). The different expression of protein isoforms in the two types of photoreceptors is likely to contribute to the unique release efficacy of their ribbon synapses. Furthermore, these examples highlight the high sequence coverage as a key feature of our proteome resource, rather than the sheer number of identified proteins. This feature is essential for uncovering further photoreceptor type–specific protein isoforms.

## Discussion

### The Mouse Photoreceptor Proteome Resource: Strengths and Limitations

**Figure 7.**
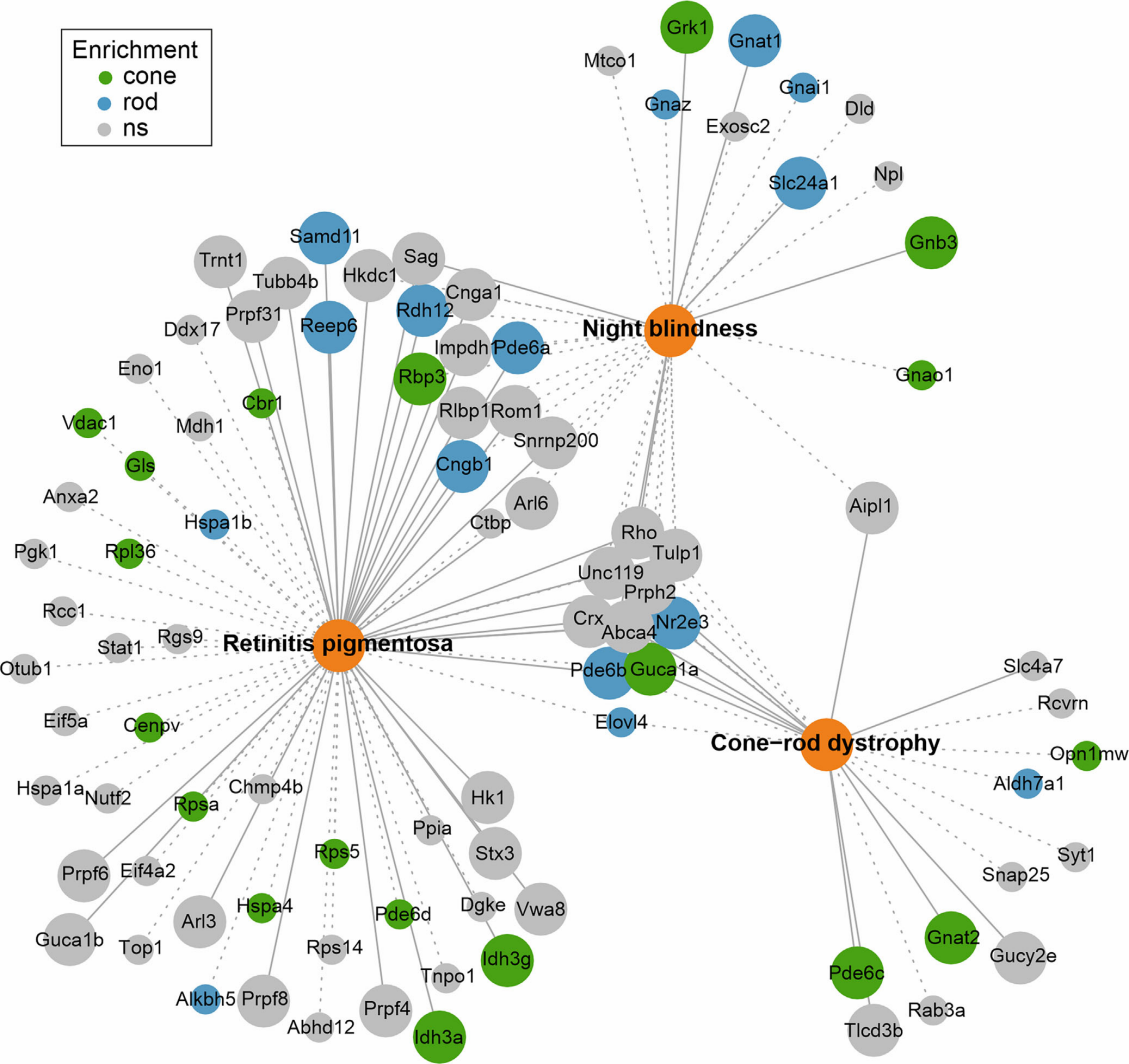
Disease-associated photoreceptor proteins. Proteins detected in our proteome resource and the disease association of respective human orthologs according to the Autoseed R package (malacards database)^89^ (*small circles*; *dotted lines*) and RetNet, the Retinal Information Network^90^ (*big circles*; *continuous lines*). Rod and cone photoreceptor–enriched proteins are highlighted in *blue* and *green*, respectively. Proteins were considered significantly enriched if −log_10_[*q*-value] > 2 and log_2_FC < −2 (cones) and/or log_2_FC > 2 (rods). Gene names were used instead of protein names to improve readability.

The mouse retina is a genetically accessible model system that is used for basic and translational research studies. The rod and cone photoreceptors are the highly specialized sensory neurons in the retina that initiate visual perception. Due to the numerous cellular processes involved in transducing and transmitting light signals and their high energy consumption, photoreceptors are highly susceptible to damage, leading to visual impairment or even blindness. Our study aimed to create a quantitative proteome resource for rod and cone photoreceptors in the mouse retina. This resource can be used to identify and assign proteins to the photoreceptor types in the healthy and diseased retina (Fig. 7; Supplementary Data S1).

In the mouse retina, approximately 97% of photoreceptors are rod photoreceptors, whereas cone photoreceptors account for only 3%.^4^ The current proteome resources available for the mouse retina do not distinguish between the two photoreceptor types and therefore predominantly reflect the protein composition of rod photoreceptors.^31^^,^^34^^,^^35^ A strength of our photoreceptor proteome resource is that it accounts for the different numbers of cells and amounts of cellular protein, enabling valid comparative analyses of the two types of photoreceptors.

One weakness to note is that the trituration-based sorting strategy enriched photoreceptor perikarya (Fig. 1A). We hypothesize that synaptic terminals retracted to some extent during the sorting process and that the OSs ripped off at the fragile connecting cilium between the OSs and ISs were lost. This agrees well with the presence of presynaptic and active zone proteins in our proteome resource, while rhodopsin, the most abundant protein in rod photoreceptor OS,^78^ was detected but not significantly enriched in rod photoreceptors (Supplementary Data S1). In contrast, the phototransduction proteins tansducin and phosducin, present in the OSs and ISs,^6^ showed strong enrichment in rod photoreceptors (Figs. 1, 2). These findings demonstrate an uneven loss of subcellular compartments, which should be considered when using this proteome resource. Furthermore, this loss may also differ between the two types of photoreceptors. This is indicated by the strongly enriched cone photoreceptor opsins, in contrast to the not significantly enriched rhodopsin (Supplementary Data S1).

Regarding sex-specific proteome differences, we note that only male mice were used in this study to avoid potential confounding effects of the female estrous cycle.^79^ However, since sex has been shown to impact the pathogenesis of neurodegenerative diseases and retinal disorders,^80^^,^^81^ future studies on the photoreceptor proteome from female mice are necessary to identify proteins that may be involved in the onset and/or progression of retinal diseases in a sex-dependent manner.

One strength of our proteome resource is its ability to characterize the protein composition of the two types of photoreceptors in great detail (Figs. 2^3^–4). Analyzing identified tryptic peptides can differentiate highly similar protein isoforms (Fig. 6; Supplementary Figure S6; Supplementary Data S3), which is important because antibodies often target shared epitopes and cannot distinguish between protein isoforms. Undoubtedly, proteomics has a lower analytical depth than transcriptomics, resulting in fewer candidates being identified (Fig. 1E compared to Supplementary Fig. S1A). Nevertheless, our proteome resource enables the quantitative analysis of protein constituents and molecular networks, which is not necessarily reflected by the corresponding transcript levels.^26^^,^^27^ scRNA-seq data sets are a powerful tool for confirming expression at the transcript level (Supplementary Figs. S1, S2). In complex neuronal tissues, such as the retina, the synergistic integration of the proteome and transcriptome data is especially desirable.

### PALS2/MPP6, a Novel Pan-Cone Photoreceptor Marker

Through an unbiased differential enrichment analysis of the proteomic profiles of rod and cone photoreceptors, we identified PALS2/MPP6 as a novel pan-cone photoreceptor protein (Figs. 4, 5; Supplementary Figs. S1–S5). PALS2/MPP6 belongs to the family of membrane-associated guanylate kinases and is implicated in receptor clustering and synaptic function.^66^^,^^82^ It has been detected previously in the mouse retina by mass spectrometry, showing significant enrichment in the ONL compared to the inner and outer photoreceptor segments.^35^ The findings of Todorova et al.^35^ corroborate our observation of PALS2/MPP6 immunoreactivity in the ONL and OPL, as well as confirm its absence in cone photoreceptor inner and outer segments (Fig. 5). In the human retina, Pals2/Mpp6 is also enriched in cone photoreceptors at the transcript level,^83^^,^^84^ and it has been detected by mass spectrometry in the juxta-macular region.^28^ We introduce PALS2/MPP6 as a new cone photoreceptor marker, in addition to the known markers, such as cone opsins,^85^ peanut agglutinin,^86^ cone arrestin,^87^ and cone transducin.^88^ Furthermore, our findings pave the way for further investigations into the retinal function of PALS2/MPP6.

### Insights Into Photoreceptor Type–Specific Protein Isoforms

An apparent strength of our mouse retina proteome resource is its potential for assessing photoreceptor type–specific protein isoforms. For example, detailed analyses of tryptic peptides revealed that the predominant PALS2/MPP6 isoform in cone photoreceptors is the noncanonical PALS2α (Fig. 6). Because our research interests lie in the proteins of ribbon synapses, we employed the same strategy to evaluate the photoreceptor type–specific distribution of PSD95β (Supplementary Fig. S6) and SNAP25a/b (Fig. 6). At the transcript level, PSD95β is the predominant isoform in the mouse retina, while PSD95α is only moderately expressed.^74^^,^^75^ PSD95β contains an additional L27 domain that is essential for synaptic clustering^73^ and the correct targeting of the plasma membrane Ca^2+^ pump complex to the photoreceptor synapse.^74^ Due to the lack of isoform-specific antibodies, it was not possible to discriminate between the two splice variants at the protein level.^75^ However, our proteome resource now provides clear evidence for PSD95β in rod photoreceptors, whereas the PSD95β-specific tryptic peptide was not detected in our cone photoreceptor samples. As with PSD95α/β, SNAP25a and SNAP25b have been shown to be present in the mouse retina at the transcript level.^77^ We can now confirm this finding at the protein level using our proteome resource.

To evaluate whether the coverage of retinal protein isoforms is a unique feature of our proteome resource, we reanalyzed the recent data set by Todorova et al.^35^ (PXD034057), which was generated by retinal layer–specific proteomics of photoreceptor inner and outer segments and the ONL, using a database search against a conventional SwissProt mouse database only containing canonical entries. By applying the same data analysis strategy as described by Todorova et al.^35^ (see Materials and Methods for details), but with the protein sequence file for database search used by us in the present study, we confirmed the presence of RIBEYE and STX3B but did not obtain mass spectrometric evidence for isoform-specific peptides that would allow us to assign the noncanonical isoforms PSD95β, SNAP25a, or PALS2α.

We are aware of a potentially limited proteome depth of our data set due to the use of a previous-generation mass spectrometer. However, we suggest that the combination of the specificity of photoreceptor enrichment by FACS—instead of layer-specific proteomics—and the high protein sequence coverage typically obtained by DIA mass spectrometry—instead of data-dependent acquisition—makes our proteome resource a versatile tool for exploring photoreceptor type–specific proteins and protein isoforms. In conclusion, we expect our photoreceptor proteome resource to be a valuable database tool that will foster future retinal research.

## Supplementary Material

Supplement 1

Supplement 2

Supplement 3
