# Supplementary material for: A Mouse Photoreceptor Proteome Resource Identifies PALS2/MPP6 as a Novel Pan-Cone Photoreceptor Marker
Source: Invest Ophthalmol Vis Sci. 2025 Dec 15;66(15):44. doi: 10.1167/iovs.66.15.44 (PMC12710781; doi:10.1167/iovs.66.15.44)
Supplement: Supplement 1 [file iovs-66-15-44_s001.pdf]

# Supplementary Material

## Supplementary Data

**Data S1.** Label-free quantification of proteins in retina lysate and FACS-sorted photoreceptors.

**Data S2**. Over-representation analyses (ORA) of rod and cone photoreceptor-enriched proteins.

**Data S3.** Peptide identification and quantification data after analysis of the raw search results by ISOQuant software.

## Supplementary Tables

**Table S1.** General information about analyzed samples.

| animal | sex | age (weeks) | sorted cones | sorted rods |
|---|---|---|---|---|
| 1 | male | 10 | 40 000 | 120 000 |
| 2 | male | 10 | 30 000 | 90 000 |
| 3 | male | 10 | 30 000 | 90 000 |
| 4 | male | 10 | 30 000 | 90 000 |
| 5 | male | 11 | 40 000 | 120 000 |
| 6 | male | 11 | 40 000 | 120 000 |

**Table S2.** Analysis of photoreceptor purity. Inspection of non-photoreceptor canonical markers for major retinal cell classes in the proteome resource. Markers mentioned in the Results (bold) and additional markers according to the Mouse Retina Cell Atlas (MRCA)[1]. BC, bipolar cell; HC, horizontal cell; MG, Muller glia cell; RGC, retinal ganglion cell; RPE, retinal pigment epithelium.

| Cell types | Gene | Protein | Uniprot ID | Found in dataset | Not in dataset |
|---|---|---|---|---|---|
| **HC** | **Calb1** | **CALB1** | **P12658** | | **X** |
| **BC** | **CaBP5** | **CABP5** | **Q9JLK3** | | **X** |
| **AC, HC, MG, RGC, Astrocytes** | **Pax6** | **PAX6** | **P63015** | | **X** |
| **RGC** | **Rbpms** | **RBPMS** | **Q9WVB0** | | **X** |
| **MG, Astrocytes** | **Gfap** | **GFAP** | **P03995** | **X** | |
| BC | Vsx2 | VSX2 | Q61412 | | X |
| HC | Onecut1 | HNF6 | O08755 | | X |
| MG, Astrocytes | Slc1a3 | EAA1 | P56564 | X | |
| Microglia | Cd74 | HG2A | P04441 | | X |
| Endothelial | Pecam1 | PECA1 | Q08481 | | X |
| Pericyte | Pdgfrb | PGFRB | P05622 | | X |
| RPE | Rpe65 | RPE65 | Q91ZQ5 | | X |

**Table S3.** Cone and rod photoreceptor-enriched proteins of the visual perception pathway. Supporting information for Figure 2. Gene names were used instead of protein names to improve readability. PhR, photoreceptor.

| PhR | Entry | Description | $\log_2$(FC) | $-\log_{10}$(q) |
| --- | --- | --- | --- | --- |
| Cone | Opn1mw | Medium-wave-sensitive opsin 1 | -6.96 | 8.88 |
| | Arr3 | Arrestin-C | -6.22 | 8.46 |
| | Opn1sw | Short-wave-sensitive opsin 1 | -5.14 | 8.58 |
| | Gnat2 | Guanine nucleotide-binding protein G(t) subunit alpha-2 | -4.75 | 10.11 |
| | Slc4a10 | Sodium-driven chloride bicarbonate exchanger | -3.74 | 7.03 |
| | Cplx3 | Complexin 3 | -3.63 | 6.74 |
| | Pde6c | Cone cGMP-specific 3'5'-cyclic phosphodiesterase subunit alpha | -2.47 | 8.30 |
| Rod | Gnat1 | Guanine nucleotide-binding protein G(t) subunit alpha-1 | 2.18 | 9.91 |
| | Pdc | Phosducin | 2.67 | 6.01 |
| | Nxnl2 | Nucleoredoxin-like protein 2 | 2.87 | 6.20 |
| | Reep6 | Receptor expression-enhancing protein 6 | 3.04 | 8.76 |
| | Cngb1 | Cyclic nucleotide-gated channel beta-1 | 4.77 | 4.05 |
| | Rorb | Nuclear receptor ROR-beta | 5.39 | 7.69 |
| | Pde6b | Rod cGMP-specific 3' 5'-cyclic phosphodiesterase subunit beta | 5.41 | 9.70 |
| | Slc24a1 | Sodium/potassium/calcium exchanger 1 | 5.47 | 8.37 |

**Table S4.** Top cone and rod photoreceptor-enriched proteins according to $-\log_{10}$[q-value] > 8 and $\log_2$FC > 5/< −5. Supporting information for Figure 4. Gene names were used instead of protein names to improve readability.

| PhR | Entry | Description | $\log_2$(FC) | $-\log_{10}$(q) |
|---|---|---|---|---|
| Cone | Gulo | L-gulonolactone oxidase | -7.80 | 9.69 |
| | Ahcyl2 | Putative adenosylhomocysteinase 3 | -7.12 | 9.50 |
| | Opn1mw | Medium-wave-sensitive opsin 1 | -6.96 | 8.88 |
| | Arr3 | Arrestin-C | -6.22 | 8.46 |
| | Prkar1a | cAMP-dependent protein kinase type I-alpha regulatory subunit | -5.90 | 9.70 |
| | Pals2/Mpp6 | Protein PALS2 (Protein Associated with Lin7 2) | -5.63 | 9.91 |
| | Opn1sw | Short-wave-sensitive opsin 1 | -5.14 | 8.58 |
| Rod | Hspa1b | Heat shock 70 kDa protein 1B | 8.17 | 10.11 |
| | Ddx39a | ATP-dependent RNA helicase DDX39A | 6.19 | 9.15 |
| | Sec14l2 | SEC14-like protein 2 | 5.20 | 9.46 |
| | Nt5e | 5'-nucleotidase | 5.82 | 9.70 |
| | Pde6b | Rod cGMP-specific 3'_5'-cyclic phosphodiesterase subunit beta | 5.41 | 9.70 |
| | Faim | Fas apoptotic inhibitory molecule 1 | 5.35 | 8.33 |
| | Ddx3y | ATP-dependent RNA helicase DDX3Y | 5.27 | 8.14 |
| | Slc24a1 | Sodium/potassium/calcium exchanger 1 | 5.47 | 8.37 |

**Table S5.** Peptide identification details for PALS2/MPP6. Columns show from left to right: numbering of amino acids according to the PALS2α sequence (Uniprot Q9JLB0-2); isoform specificity (PALS2α, PALS2β or shared); peptide sequence (carboxamidomethylated Cys as fixed modification); calculated mass of the singly protonated peptide, observed mass of the singly protonated peptide; peptide mass deviation in ppm; PLGS score; number of b–y fragment ions; root mean square fragment mass deviation in ppm. For all observed values, the minimum-maximum range from the six cone photoreceptor samples is shown.

| Peptide | Isoform specificity | Sequence | $[M+H]^+_{calc}$ | $[M+H]^+_{obs}$ (min-max) | ΔM (ppm) (min-max) | PLGS Score (min-max) | b-y (min-max) | RMS (ppm) (min-max) |
|---|---|---|---|---|---|---|---|---|
| 86-102 | shared | EPHFQSLLEAHDIVASK | 1920.9814 | 1920.9803-1920.9841 | -0.53-1.43 | 6.74-7.12 | 10-23 | 9.18-13.35 |
| 129-134 | shared | ILGIHK | 680.4454 | 680.4432-680.4459 | -3.20-0.73 | 6.78-7.38 | 2-3 | 1.53-9.66 |
| 136-145 | shared | AGEPLGVTFR | 1046.5629 | 1046.5600-1046.5632 | -2.69-0.29 | 6.96-7.44 | 5-8 | 2.62-8.42 |
| 146-155 | shared | VENNDLVIAR | 1142.6165 | 1142.6136-1142.6160 | -2.40-(-0.38) | 7.43-7.80 | 15-19 | 5.98-9.61 |
| 156-164 | shared | ILHGGMIDR | 1011.5404 | 1011.5393-1011.5533 | -1.10-12.78 | 6.51-6.75 | 2-6 | 11.58-6.65 |
| 165-175 | shared | QGLLHVGDIIK | 1192.7048 | 1192.7028-1192.7054 | -1.67-0.44 | 5.58-6.54 | 4-10 | 7.48-10.35 |
| 188-194 | shared | ELQELLK | 872.5088 | 872.5080-872.5102 | -0.82-1.67 | 7.09-7.72 | 7-14 | 7.19-13.00 |
| 195-203 | shared | NISGSVTLK | 918.5255 | 918.5230-918.5265 | -2.72-1.12 | 6.76-7.49 | 3-7 | 3.74-13.97 |
| 204-209 | shared | ILPSYR | 748.4352 | 748.4336-748.4350 | -2.19-(-0.30) | 6.99-7.03 | 2-5 | 11.72-15.98 |
| 210-220 | PALS2α | DTITPQQVFVK | 1275.6943 | 1275.6935-1275.6954 | -0.63-0.82 | 6.93-7.61 | 8-15 | 5.86-12.43 |
| 221-236 | shared | CHFDYNPFNDNLIPCK | 2053.8896 | 2053.8860-2053.8940 | -1.80-2.17 | 7.00-7.68 | 9-21 | 8.39-11.47 |
| 245-253 | shared | GEILQIVNR | 1041.6051 | 1041.6017-1041.6040 | -3.25-(-1.06) | 7.48-8.25 | 10-12 | 4.86-7.44 |
| 254-265 | shared | EDPNWWQASHVK | 1496.6917 | 1496.6857-1496.6956 | -4.03-2.58 | 7.16-7.68 | 10-18 | 7.22-12.89 |
| 266-281 | shared | EGGSAGLIPSQFLEEK | 1661.8381 | 1661.8363-1661.8411 | -1.06-1.81 | 7.74-8.38 | 20-26 | 5.40-9.47 |
| 288-303 | shared | DWDNSGPFCGTISNK | 1697.7224 | 1697.7227-1697.7279 | 0.14-3.23 | 6.28-7.03 | 7-10 | 8.86-16.08 |
| 339-350 | shared | TLVLIGAQGVGR | 1183.7157 | 1183.7133-1183.7166 | -2.07-0.70 | 7.68-8.06 | 12-14 | 5.89-8.22 |
| 357-364 | shared | FIVLNPAR | 929.5567 | 929.5543-929.5573 | -2.55-0.59 | 7.33-7.68 | 7-11 | 8.07-10.83 |
| 365-374 | shared | FGTTVPFTSR | 1112.5735 | 1112.5714-1112.5760 | -1.84-2.38 | 6.67-7.61 | 7-14 | 6.29-11.93 |
| 403-417 | shared | YLEHGEYEGNLYGTK | 1772.8126 | 1772.8123-1772.8177 | -0.19-2.87 | 7.39-7.73 | 16-22 | 8.09-11.21 |
| 418-429 | shared | IDSILEVVQTGR | 1329.7373 | 1329.7332-1329.7394 | -3.08-1.64 | 7.41-7.95 | 18-26 | 4.84-9.04 |
| 430-441 | shared | TCILDVNPQALK | 1371.7301 | 1371.7195-1371.7247 | -7.71-(-3.90) | 6.99-7.32 | 7-12 | 6.42-10.63 |
| 469-478 | shared | AVVDAGITTK | 974.5517 | 974.5441-974.5481 | -7.79-(-3.72) | 7.23-7.47 | 9-9 | 5.10-8.25 |
| 479-486 | shared | LLTDSDLK | 904.4986 | 904.4968-904.4984 | -2.02-(-0.17) | 7.01-7.42 | 4-6 | 3.71-10.07 |
| 498-514 | shared | AYNHYFDLIIVNDNLDK | 2067.0183 | 2067.0186-2067.0250 | 0.15-3.24 | 6.16-6.87 | 6-9 | 8.32-15.43 |
| 519-525 | shared | LQTAIEK | 802.4669 | 802.4654-802.4664 | -1.86-(-0.63) | 6.98-7.55 | 5-7 | 8.20-10.72 |

**Table S6.** Peptide identification details for SNAP25. Columns show from left to right: numbering of amino acids according to the SNAP25b sequence (Uniprot P60879-1); isoform specificity (SNAP25a, SNAP25b or shared); peptide sequence; calculated mass of the singly protonated peptide, observed mass of the singly protonated peptide; peptide mass deviation in ppm; PLGS score; number of b–y fragment ions; root mean square fragment mass deviation in ppm. For all observed values, the minimum-maximum range from the six cone and six rod photoreceptor samples is shown.

| Peptide | Isoform specificity | Sequence | $[M+H]^+_{calc}$ | $[M+H]^+_{obs}$ (min-max) | ΔM (ppm) (min-max) | PLGS Score (min-max) | b-y (min-max) | RMS (ppm) (min-max) |
|---|---|---|---|---|---|---|---|---|
| 9-16 | shared | NELEEMQR | 1048.4728 | 1048.4733-1048.4803 | 0.46-7.14 | 6.36-7.14 | 2-6 | 6.68-12.72 |
| 18-30 | shared | ADQLADESLESTR | 1434.6707 | 1434.6690-1434.6866 | -1.26-11.11 | 5.52-7.62 | 3-12 | 7.34-14.87 |
| 32-40 | shared | MLQLVEESK | 1076.5656 | 1076.5596-1076.5650 | -5.57-(-0.64) | 6.26-6.77 | 2-4 | 3.63-16.02 |
| 46-59 | SNAP25a | TLVMLDEQGEQLDR | 1646.8054 | 1646.8033-1646.8160 | -1.21-6.46 | 5.74-6.44 | 3-4 | 6.61-13.64 |
| 46-59 | SNAP25b | TLVMLDEQGEQLER | 1660.8210 | 1660.8187-1660.8260 | -1.43-2.95 | 6.33-7.43 | 7-17 | 7.31-14.93 |
| 60-69 | SNAP25b | IEEGMDQINK | 1176.5565 | 1176.5538-1176.5569 | -2.30-0.30 | 6.67-7.29 | 4-9 | 7.56-10.41 |
| 77-83 | SNAP25b | NLTDLGK | 760.4199 | 760.4183-760.4190 | -2.17-(-1.30) | 5.80-7.42 | 2-9 | 10.30-14.90 |
| 104-119 | shared | AWGNNQDGVVASQPAR | 1669.8041 | 1669.8007-1669.8069 | -2.09-1.65 | 6.98-8.12 | 14-23 | 8.30-12.56 |
| 125-135 | shared | EQMAISGGFIR | 1208.6093 | 1208.6068-1208.6099 | -1.98-0.54 | 6.91-7.74 | 8-17 | 5.80-9.90 |
| 143-161 | shared | ENEMDENLEQVSGIIGNLR | 2160.0237 | 2160.0134-2160.0256 | -4.7413-0.8899 | 6.20-7.16 | 4-20 | 9.38-13.64 |
| 162-176 | shared | HMALDMGNEIDTQNR | 1744.7740 | 1744.7690-1744.7823 | -2.8888-4.6972 | 5.66-6.90 | 2-10 | 3.96-13.71 |

**Table S7.** R and python packages. Python version 3.10.14, *used with R version 4.4.2, all other packages with R version 4.3.2.

| R packages | |
|---|---|
| **Name[Reference]** | **Version** |
| Autoseed[2] | 0.1.0 |
| clusterProfiler[3] | 4.16.0 |
| DESeq2[4] | 1.42.1 |
| dplyr[5] | 1.1.4 |
| factoextra[6] | 1.0.7 |
| FactoMineR[7] | 2.11 |
| ggplot2[8] | 3.5.1 |
| ggpubr[9] | 0.6.0 |
| ggrepel[10] | 0.9.6 |
| gprofiler2[11] | 0.2.3 |
| igraph[12] | 2.1.4 |
| limma*[13] | 3.62.1 |
| missForest[14] | 1.5 |
| org.Mm.eg.db[15] | 3.18.0 |
| pheatmap[16] | 1.0.13 |
| qvalue*[17] | 2.38.0 |
| RColorBrewer[18] | 1.1-3 |
| readxl[19] | 1.4.5 |
| tibble[20] | 3.2.1 |
| tidyr[21] | 1.3.1 |
| **Python packages** | |
| **Name** | **Version** |
| numpy[22] | 1.24.3 |
| scanpy[23] | 1.10.1 |
| pandas[24] | 2.2.2 |

## Supplementary Figures

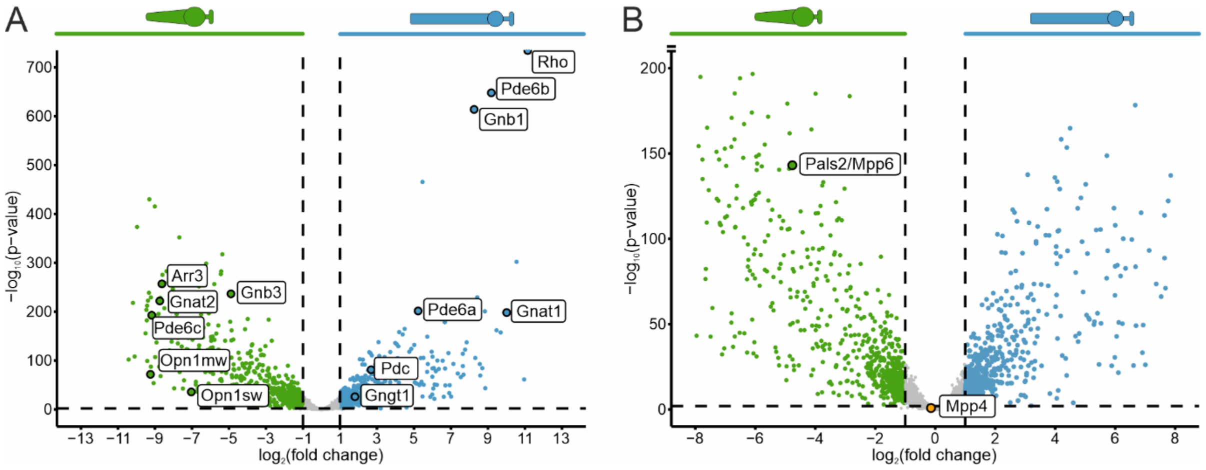


**Figure S1**. Photoreceptor-specific transcript profiling of cone and rod photoreceptor markers and Pals2/Mpp6 and Mpp4. Supporting information for Figures 1 and 4. **A,B**, Pseudobulk analyses based on mouse retina cell atlas (MRCA) scRNA-seq data set by Li et al. (2024)[1] showing the expression of rod- and cone photoreceptor-specific markers (**A**), and Mpp4 and Pals2/Mpp6 (**B**).

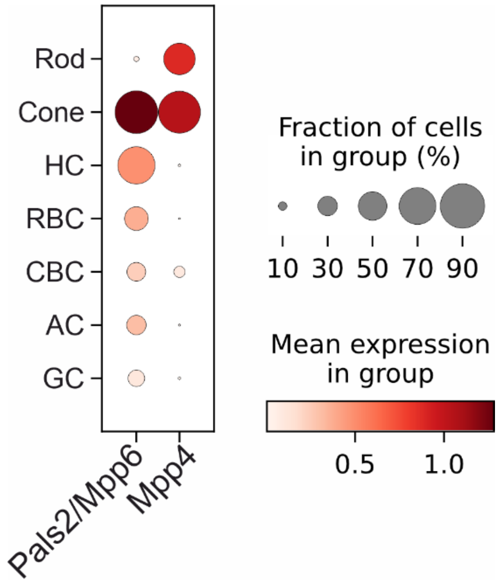


**Figure S2**. Expression of Pals2/Mpp6 and Mpp4 in major cell types of mouse retinae. Visualized mean expression based on scRNA-seq data set by Li et al. (2024)[1] HC, horizontal cells; RBC, rod bipolar cells; CBC, cone bipolar cells; AC amacrine cells; GC, ganglion cells.

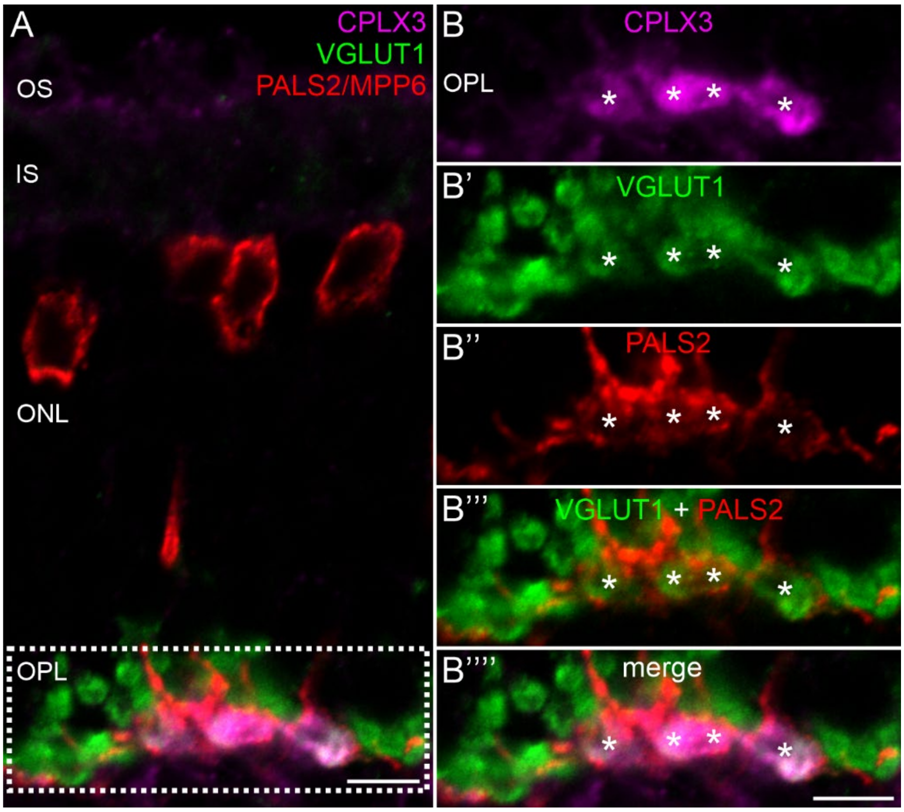


**Figure S3**. PALS2/MPP6 in cone photoreceptor terminals. **A-B'''',** Confocal micrographs of vertical cryostat sections through mouse retinae stained with anti-Complexin 3 (CPLX3), anti-vesicular glutamate transporter 1 (VGLUT1), and anti-PALS2/MPP6 antibodies. Higher magnification confocal micrographs of the outer plexiform layer (OPL) showing PALS2/MPP6-positive cone photoreceptor terminals ($CPLX3^+$/$VGLUT1^+$/$PALS2/MPP6^+$; asterisks) and PALS2/MPP6-negative rod photoreceptor terminals ($CPLX3^–$/$VGLUT1^+$/$PALS2/MPP6^–$). OS, outer segments; IS, inner segments; ONL, outer nuclear layer. Scale bars = 5 µm in (**A**) and (**B''''**).

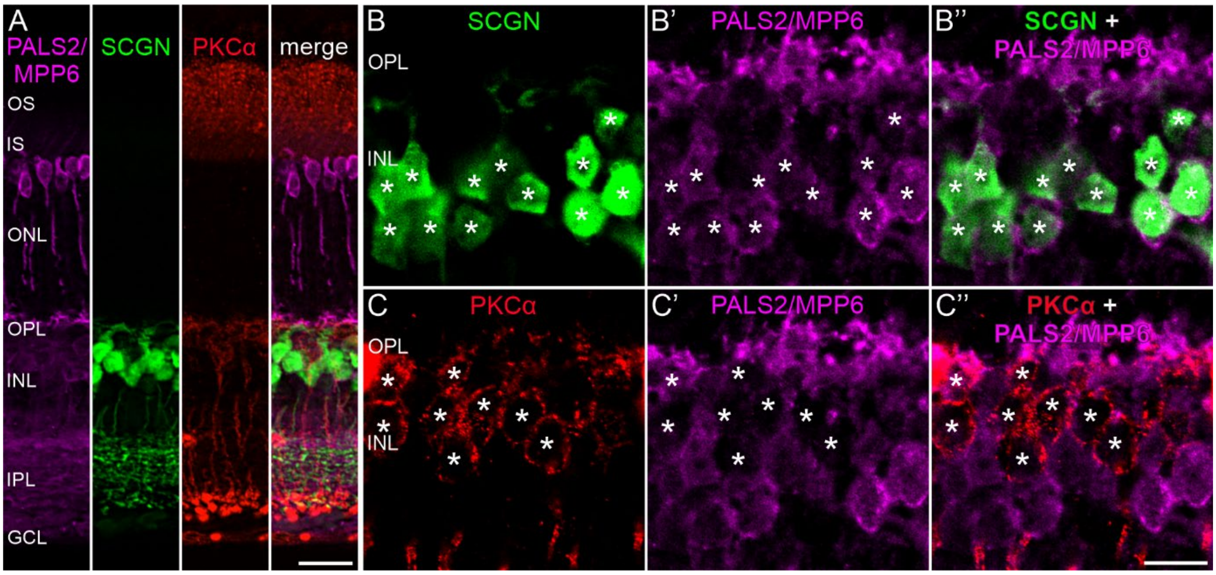


**Figure S4**. PALS2/MPP6 in cone bipolar cells. **A-C'',** Fluorescence micrographs of vertical cryostat sections through mouse retinae stained with anti-PALS2/MPP6, anti-Secretagogin (SCGN), and anti-Protein kinase C alpha (PKCα) antibodies. Higher magnification confocal micrographs of the outer plexiform layer (OPL) and inner nuclear layer (INL) showing stained cell bodies highlighted with asterisks for SCGN (**B-B''**) and PKCα (**C-C''**). OS, outer segments; IS, inner segments; ONL, outer nuclear layer; OPL, outer plexiform layer; GCL, ganglion cell layer. Scale bar = 20 μm in (**A**) and 10 μm in (**C''**) for (**B-C''**).

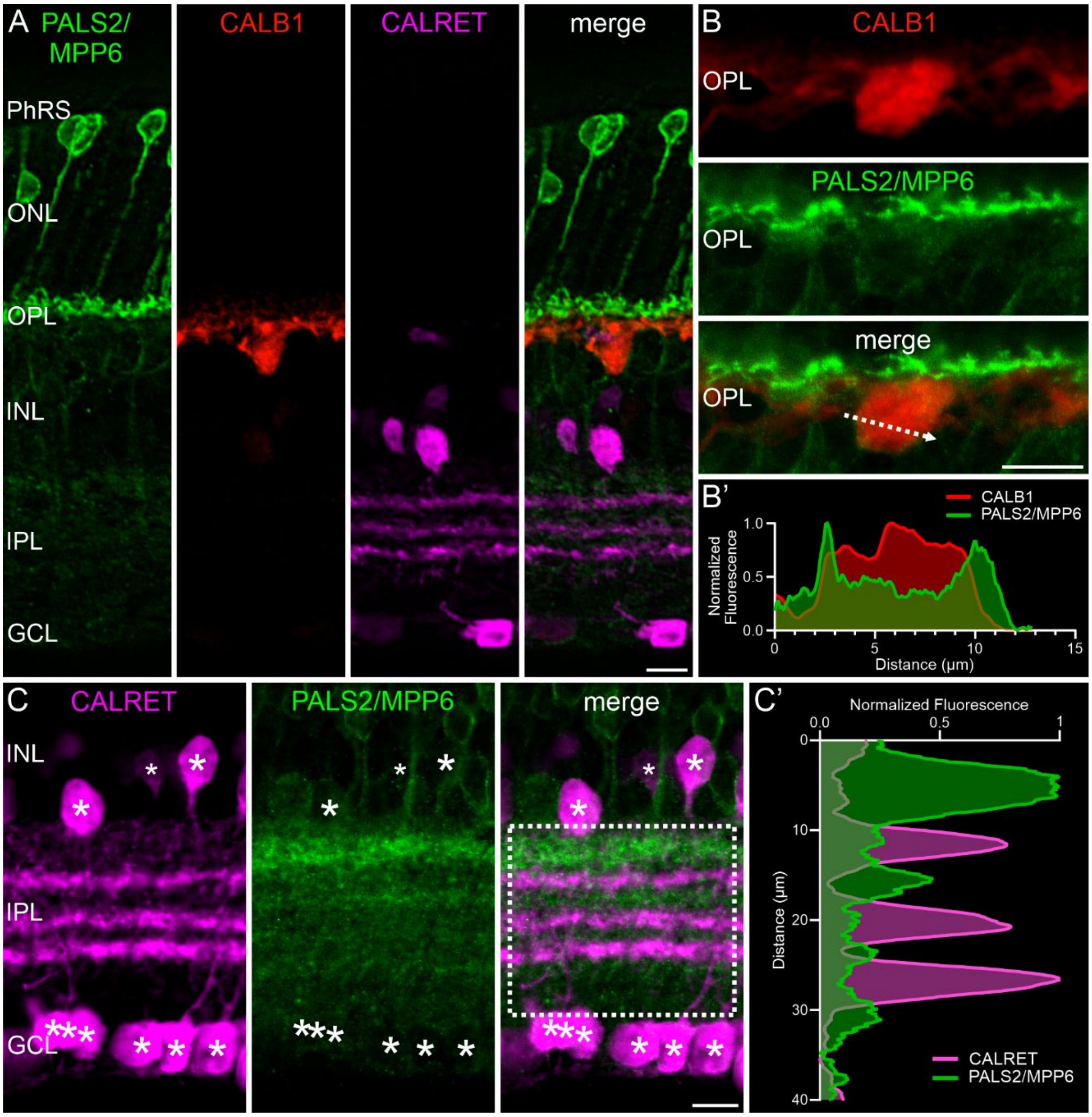


**Figure S5**. PALS2/MPP6 in horizontal cells. **A-C',** Fluorescence micrographs of vertical cryostat sections through mouse retinae stained with anti-PALS2/MPP6, anti-Calretinin (CALRET), and anti-Calbindin (CALB1) antibodies. Higher-power confocal micrographs of the outer plexiform layer (OPL) showing a CALB1-positive cell body (**B**). Line profile through CALB1-positive cell (**B'**) as indicated by white dotted line in (**B**). Higher magnification confocal micrographs of the inner nuclear layer (INL), inner plexiform layer (IPL), and ganglion cell layer (GCL) with CALRET-positive somata highlighted by asterisks (**C**). Area intensity profile of the IPL (**C'**) indicated by the white dotted frame in (**C**). PhRS, photoreceptor segments; ONL, outer nuclear layer. All scale bars = 10 µm.

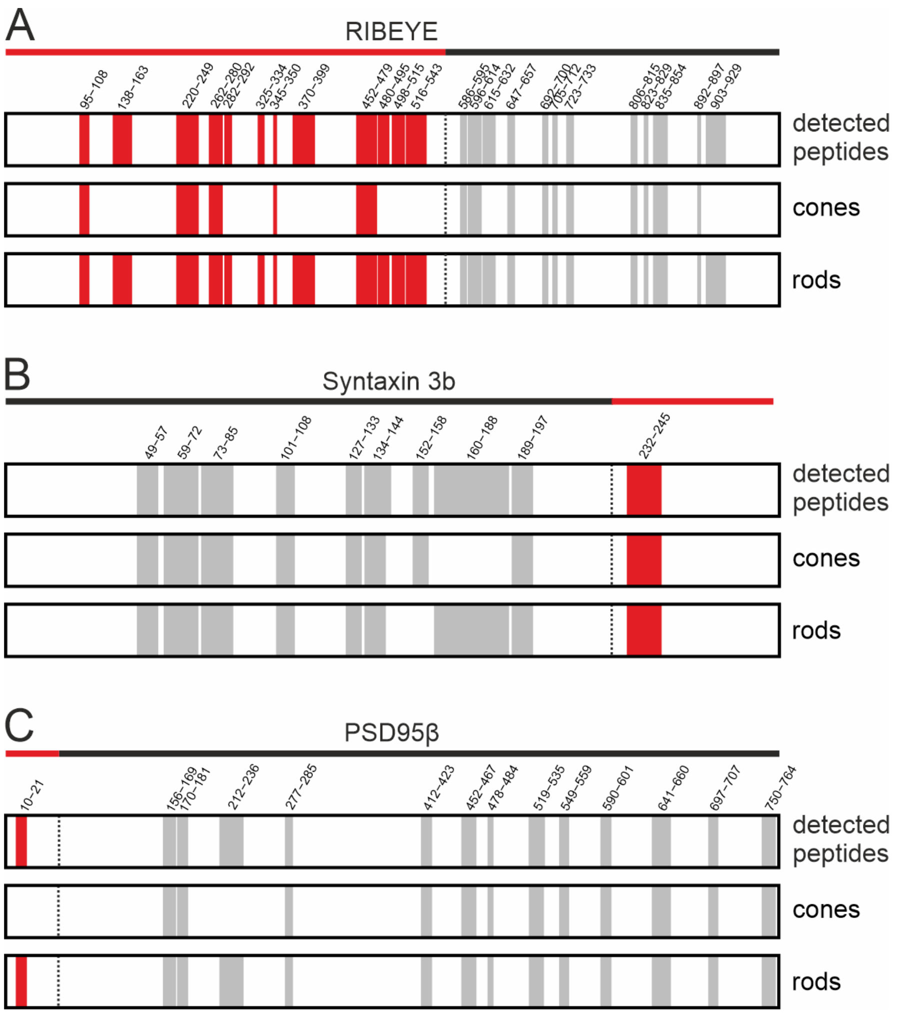


**Figure S6**. Identification of the retina-specific protein isoforms RIBEYE, Syntaxin 3b and PSD95β in cone and rod photoreceptors. Supporting information for Figure 6. **A-C**, Identified tryptic peptides (n ≥ 3 per group) for RIBEYE (**A**), Syntaxin 3b (**B**) and PSD95β. Isoform-specific peptides are highlighted in red.

## Supplementary Scripts

**Script S1.** R Notebook for principal component analysis (PCA) and correlation heatmap. Supporting information for Figure 1.

**Script S2.** R Notebook for volcano plots. Supporting information for Figures 1, 3, 4.

**Script S3.** R Notebook for protein abundance analyses. Supporting information for Figures 1, 3, 4.

**Script S4.** R Notebook for over-representation analyses (ORA). Supporting information for Figure 2.

**Script S5.** R Notebook for tryptic peptide analyses. Supporting information for Figure 6 and Supplementary Figure S6.

**Script S6.** R Notebook for disease association analyses. Supporting information for Figure 7.

**Script S7.** Jupyter Notebook for pseudobulk analyses. Supporting information for Supplementary Figure S1.

**Script S8.** R Notebook for volcano plot after pseudobulking. Supporting information for Supplementary Figure S1.

**Script S9.** Jupyter Notebook for scRNA-Seq dotplot. Supporting information for Supplementary Figure S2.

**Literature**


1. Li J, Choi J, Cheng X, et al. Comprehensive single-cell atlas of the mouse retina. *iScience*. 2024;27(6):109916. doi:10.1016/j.isci.2024.109916

2. Wu J, Li X. Autoseed: Retrieve Disease-Related Genes from Public Sources. Published online 2020. https://CRAN.R-project.org/package=Autoseed

3. Wu T, Hu E, Xu S, et al. clusterProfiler 4.0: A universal enrichment tool for interpreting omics data. *The Innovation*. 2021;2(3):100141. doi:10.1016/j.xinn.2021.100141

4. Love MI, Huber W, Anders S. Moderated estimation of fold change and dispersion for RNA-seq data with DESeq2. *Genome Biology*. 2014;15(12):550. doi:10.1186/s13059-014-0550-8

5. Wickham H, François R, Henry L, Müller K, Vaughan D. dplyr: A Grammar of Data Manipulation. Published online 2023. https://CRAN.R-project.org/package=dplyr

6. Kassambara A, Mundt F. factoextra: Extract and Visualize the Results of Multivariate Data Analyses. Published online 2020. https://CRAN.R-project.org/package=factoextra

7. Lê S, Josse J, Husson F. FactoMineR: An R Package for Multivariate Analysis. *Journal of Statistical Software*. 2008;25:1-18. doi:10.18637/jss.v025.i01

8. Wickham H. *Ggplot2: Elegant Graphics for Data Analysis*. Second edition. Springer international publishing; 2016.

9. Kassambara A. ggpubr: “ggplot2” Based Publication Ready Plots. Published online 2023. https://CRAN.R-project.org/package=ggpubr

10. Slowikowski K. ggrepel: Automatically Position Non-Overlapping Text Labels with “ggplot2.” Published online 2024. https://CRAN.R-project.org/package=ggrepel

11. Kolberg L, Raudvere U, Kuzmin I, Vilo J, Peterson H. gprofiler2 -- an R package for gene list functional enrichment analysis and namespace conversion toolset g:Profiler. *F1000Res*. 2020;9:ELIXIR-709. doi:10.12688/f1000research.24956.2

12. Csárdi G, Nepusz T, Traag V, et al. igraph: Network Analysis and Visualization in R. Published online 2025. <https://CRAN.R-project.org/package=igraph>

13. Ritchie ME, Phipson B, Wu D, et al. limma powers differential expression analyses for RNA-sequencing and microarray studies. *Nucleic Acids Res*. 2015;43(7):e47. doi:10.1093/nar/gkv007

14. Stekhoven DJ, Bühlmann P. MissForest—non-parametric missing value imputation for mixed-type data. *Bioinformatics*. 2012;28(1):112-118. doi:10.1093/bioinformatics/btr597

15. Carlson M. org.Mm.eg.db: Genome wide annotation for Mouse. Published online 2023.

16. Kolde R. pheatmap: Pretty Heatmaps. Published online 2025. https://CRAN.R-project.org/package=pheatmap

17. Storey JD. The positive false discovery rate: a Bayesian interpretation and the q-value. *Ann Statist*. 2003;31(6). doi:10.1214/aos/1074290335

18. Neuwirth E. RColorBrewer: ColorBrewer Palettes. Published online 2022. https://CRAN.R-project.org/package=RColorBrewer

19. Wickham H, Bryan J. readxl: Read Excel Files. Published online 2025. https://CRAN.R-project.org/package=readxl

20. Müller K, Wickham H. tibble: Simple Data Frames. Published online 2023. https://CRAN.R-project.org/package=tibble

21. Wickham H, Vaughan D, Girlich M. tidyr: Tidy Messy Data. Published online 2024. https://CRAN.R-project.org/package=tidyr

22. Harris CR, Millman KJ, van der Walt SJ, et al. Array programming with NumPy. *Nature*. 2020;585(7825):357-362. doi:10.1038/s41586-020-2649-2

23. Wolf FA, Angerer P, Theis FJ. SCANPY: large-scale single-cell gene expression data analysis. *Genome Biology*. 2018;19(1):15. doi:10.1186/s13059-017-1382-0

24. McKinney W. Data Structures for Statistical Computing in Python. In: 2010:56-61. doi:10.25080/Majora-92bf1922-00a
